# Pharmacological interventions for prophylaxis of vestibular migraine

**DOI:** 10.1002/14651858.CD015187.pub2

**Published:** 2023-04-12

**Authors:** Katie E Webster, Afrose Dor, Kevin Galbraith, Luma Haj Kassem, Natasha A Harrington-Benton, Owen Judd, Diego Kaski, Otto R Maarsingh, Samuel MacKeith, Jaydip Ray, Vincent A Van Vugt, Martin J Burton

**Affiliations:** Cochrane ENT, Nuffield Department of Surgical SciencesUniversity of OxfordOxfordUK; Wadham CollegeUniversity of OxfordOxfordUK; Cochrane ENTNuffield Department of Surgical Sciences, University of OxfordOxfordUK; Aleppo University HospitalAleppoSyrian Arab Republic; Ménière's SocietyDorkingUK; ENT DepartmentUniversity Hospitals of Derby and Burton NHS Foundation TrustDerbyUK; National Hospital for Neurology and NeurosurgeryLondonUK; Amsterdam UMC, Vrije Universiteit Amsterdam, Department of General PracticeAmsterdam Public Health Research InstituteAmsterdamNetherlands; ENT DepartmentOxford University Hospitals NHS Foundation TrustOxfordUK; University of SheffieldSheffieldUK; Nuffield Department of Surgical SciencesUniversity of OxfordOxfordUK

**Keywords:** Adult, Female, Humans, Male, Antibodies, Monoclonal, Antibodies, Monoclonal/therapeutic use, Calcium Channel Blockers, Calcium Channel Blockers/therapeutic use, Headache, Migraine Disorders, Migraine Disorders/drug therapy, Migraine Disorders/prevention & control

## Abstract

**Background:**

Vestibular migraine is a form of migraine where one of the main features is recurrent attacks of vertigo. These episodes are often associated with other features of migraine, including headache and sensitivity to light or sound. These unpredictable and severe attacks of vertigo can lead to a considerable reduction in quality of life. The condition is estimated to affect just under 1% of the population, although many people remain undiagnosed. A number of pharmacological interventions have been used or proposed to be used as prophylaxis for this condition, to help reduce the frequency of the attacks. These are predominantly based on treatments that are in use for headache migraine, with the belief that the underlying pathophysiology of these conditions is similar.

**Objectives:**

To assess the benefits and harms of pharmacological treatments used for prophylaxis of vestibular migraine.

**Search methods:**

The Cochrane ENT Information Specialist searched the Cochrane ENT Register; Central Register of Controlled Trials (CENTRAL); Ovid MEDLINE; Ovid Embase; Web of Science; ClinicalTrials.gov; ICTRP and additional sources for published and unpublished trials. The date of the search was 23 September 2022.

**Selection criteria:**

We included randomised controlled trials (RCTs) and quasi‐RCTs in adults with definite or probable vestibular migraine comparing beta‐blockers, calcium channel blockers, antiepileptics, antidepressants, diuretics, monoclonal antibodies against calcitonin gene‐related peptide (or its receptor), botulinum toxin or hormonal modification with either placebo or no treatment. We excluded studies with a cross‐over design, unless data from the first phase of the study could be identified.

**Data collection and analysis:**

We used standard Cochrane methods. Our primary outcomes were: 1) improvement in vertigo (assessed as a dichotomous outcome ‐ improved or not improved), 2) change in vertigo (assessed as a continuous outcome, with a score on a numerical scale) and 3) serious adverse events. Our secondary outcomes were: 4) disease‐specific health‐related quality of life, 5) improvement in headache, 6) improvement in other migrainous symptoms and 7) other adverse effects. We considered outcomes reported at three time points: < 3 months, 3 to < 6 months, > 6 to 12 months. We used GRADE to assess the certainty of evidence for each outcome.

**Main results:**

We included three studies with a total of 209 participants. One evaluated beta‐blockers and the other two evaluated calcium channel blockers. We did not identify any evidence for the remaining interventions of interest.

**Beta‐blockers versus placebo**

One study (including 130 participants, 61% female) evaluated the use of 95 mg metoprolol once daily for six months, compared to placebo. The proportion of people who reported improvement in vertigo was not assessed in this study. Some data were reported on the frequency of vertigo attacks at six months and the occurrence of serious adverse effects. However, this is a single, small study and for all outcomes the certainty of evidence was low or very low. We are unable to draw meaningful conclusions from the numerical results.

**Calcium channel blockers versus no treatment**

Two studies, which included a total of 79 participants (72% female), assessed the use of 10 mg flunarizine once daily for three months, compared to no intervention. All of the evidence for this comparison was of very low certainty. Most of our outcomes were only reported by a single study, therefore we were unable to conduct any meta‐analysis. Some data were reported on improvement in vertigo and change in vertigo, but no information was available regarding serious adverse events. We are unable to draw meaningful conclusions from the numerical results, as these data come from single, small studies and the certainty of the evidence was very low.

**Authors' conclusions:**

There is very limited evidence from placebo‐controlled randomised trials regarding the efficacy and potential harms of pharmacological interventions for prophylaxis of vestibular migraine. We only identified evidence for two of our interventions of interest (beta‐blockers and calcium channel blockers) and all evidence was of low or very low certainty. Further research is necessary to identify whether these treatments are effective at improving symptoms and whether there are any harms associated with their use.

## Plain language summary

**How effective are medicines used to prevent attacks of vestibular migraine**

**Key messages**

It is not clear whether any medications are effective at preventing attacks of vestibular migraine.

There are very few studies that have assessed the possible benefits and harms of taking medication to prevent attacks. The available studies are small and the results are inconclusive.

Further work is needed in this area to help establish whether there are any treatments that may improve this condition.

**What is vestibular migraine?**

Migraine (sometimes known as 'headache migraine') is a common condition that causes recurrent headaches. Vestibular migraine is a related condition where the main symptoms are recurring episodes of severe dizziness or vertigo (a spinning sensation). These episodes are often associated with headache, or other migraine‐like symptoms (such as sensitivity to light or sound, nausea and vomiting). It is a relatively common condition, which affects up to 1 in every 100 people, and can have severe effects on day‐to‐day life.

**How is vestibular migraine treated?**

Typical treatment plans include medications to try and stop an attack of vertigo once it has started, or to improve the symptoms. In addition, people may use treatments intended to prevent attacks from starting (prophylactic or preventative treatment). There are no widely recommended treatments to prevent or manage the symptoms of a vestibular migraine attack. People are sometimes advised to take medications used to treat headache migraine. The assumption is that these medicines may also work for vestibular migraine.

**What did we want to find out?**

We wanted to find out:

‐ whether there is evidence that any medications work to prevent attacks of vestibular migraine, or reduce the symptoms when an attack occurs;

‐ whether the treatments might cause any harm.

**What did we do?**

We searched for studies including adults that compared different medications to either no treatment or placebo (dummy) treatment. We used standard methods to assess the certainty of the evidence. We rated our confidence in the evidence, based on factors such as study methods, the number of participants in them and the consistency of findings across studies.

**What did we find?**

We found three studies, which included a total of 209 people (65% female). These studies looked at two different types of medicines, to assess whether they might help to prevent vestibular migraine attacks, or help to reduce the symptoms when episodes occur.

*Beta‐blockers*

The first study looked at the use of a medicine called metoprolol, a tablet taken once daily. Metoprolol is from a group of medications known as beta‐blockers. These are often used to treat high blood pressure, but are also used to try and prevent attacks of headache migraine. It was unclear whether this treatment made any difference to the frequency of vertigo attacks, or whether it was associated with any serious harms.

*Calcium channel blockers*

Two smaller studies assessed the use of flunarizine, a tablet taken once a day. This is from the family of medications known as calcium channel blockers. Again, these medicines are commonly used to control high blood pressure, but are also used for headache migraine. It was very unclear whether people felt their symptoms had improved when taking this treatment and whether the frequency of their vertigo attacks changed. The studies did not report on serious harms of the treatment, so we do not know if there were any risks associated with taking the medication.

**What are the limitations of the evidence?**

We have very little confidence in the evidence because the three studies conducted were small. There were also some problems with the conduct of the studies, which means that the results may be unreliable. These medications are often used for other conditions, where they are known to be associated with some side effects. However, we did not identify enough information in this review to know whether these side effects are a problem when the treatments are used for vestibular migraine.

**How up‐to‐date is this evidence?**

This evidence is up‐to‐date to September 2022.

## Summary of findings

**Summary of findings 1:** Beta‐blockers compared to placebo for prophylaxis of vestibular migraine

**Beta‐blockers compared to placebo for prophylaxis of vestibular migraine**						
**Patient or population:** people with vestibular migraine **Setting:** outpatients **Intervention:** beta‐blockers **Comparison:** placebo						
**Outcomes**	Anticipated absolute effects^*^ (95% CI)		**Relative effect (95% CI)**	**№ of participants (studies)**	**Certainty of the evidence (GRADE)**	**Comments**
	**Risk with placebo**	**Risk with beta‐blockers**				
Improvement in vertigo	This outcome was not reported.					
Change in vertigo frequency Assessed with: number of vertigo attacks per month Follow‐up: range 3 months to 6 months	The mean vertigo frequency was 3.78 attacks per month	MD 0.3 attacks per month lower (1.83 lower to 1.23 higher)	—	114 (1 RCT)	⊕⊕⊝⊝ Low^1,2^	Beta‐blockers may result in little or no difference in the number of vertigo attacks at 3 to 6 months.
Serious adverse events	Study population		RR 0.63 (0.24 to 1.67)	121 (1 RCT)	⊕⊝⊝⊝ Very low^1,2,3^	The evidence is very uncertain as to whether beta‐blockers result in a change in the likelihood of serious adverse events.
	153 per 1000	96 per 1000 (37 to 255)				
*The risk in the intervention group (and its 95% confidence interval) is based on the assumed risk in the comparison group and the **relative effect** of the intervention (and its 95% CI). **CI:** confidence interval; **MD:** mean difference; **RCT:** randomised controlled trial; **RR:** risk ratio						
**GRADE Working Group grades of evidence** **High certainty:** we are very confident that the true effect lies close to that of the estimate of the effect. **Moderate certainty:** we are moderately confident in the effect estimate: the true effect is likely to be close to the estimate of the effect, but there is a possibility that it is substantially different. **Low certainty:** our confidence in the effect estimate is limited: the true effect may be substantially different from the estimate of the effect. **Very low certainty:** we have very little confidence in the effect estimate: the true effect is likely to be substantially different from the estimate of effect.						

^1^Risk of attrition bias due to substantial dropout over the course of the study. ^2^Optimal information size was not reached (taken as < 400 participants for continuous outcomes or < 300 events for dichotomous outcomes, as a rule of thumb). ^3^Wide confidence interval, which includes both the potential for considerable harm and potential benefit from the intervention.

**Summary of findings 2:** Calcium channel blockers compared to no intervention for prophylaxis of vestibular migraine

**Calcium channel blockers compared to no intervention for prophylaxis of vestibular migraine**						
**Patient or population:** people with vestibular migraine **Setting: **outpatients **Intervention:** calcium channel blockers **Comparison:** no intervention						
**Outcomes**	Anticipated absolute effects^*^ (95% CI)		**Relative effect (95% CI)**	**№ of participants (studies)**	**Certainty of the evidence (GRADE)**	**Comments**
	**Risk with no intervention**	**Risk with calcium channel blockers**				
Improvement in vertigo severity Assessed with: "marked improvement" compared to "little/no improvement" Follow‐up: range < 3 months	Study population		RR 1.45 (1.01 to 2.07)	48 (1 RCT)	⊕⊝⊝⊝ Very low^1,2,3^	Calcium channel blockers may result in an improvement in the severity of vertigo at less than 3 months follow up, but the evidence is very uncertain.
	609 per 1000	883 per 1000 (615 to 1000)				
Improvement in vertigo frequency Follow‐up: range 3 months to 6 months	Study population		RR 1.65 (0.80 to 3.41)	23 (1 RCT)	⊕⊝⊝⊝ Very low^3,4,5^	The evidence is very uncertain about the effect of calcium channel blockers on improvement in vertigo frequency after 6 months of follow‐up.
	455 per 1000	750 per 1000 (364 to 1000)				
Change in vertigo frequency Assessed with: frequency of episodes over a 3‐month period Follow‐up: range 3 months to 6 months	The mean change in vertigo frequency was 5.5 episodes in 3 months	MD 3.3 episodes in 3 months lower (5.94 lower to 0.66 lower)	—	23 (1 RCT)	⊕⊝⊝⊝ Very low^3,4,6^	Calcium channel blockers may slightly reduce the number of episodes of vertigo after 6 months follow‐up, but the evidence was very uncertain.
Serious adverse events	This outcome was not reported.					
*The risk in the intervention group (and its 95% confidence interval) is based on the assumed risk in the comparison group and the **relative effect** of the intervention (and its 95% CI). **CI:** confidence interval; **MD:** mean difference; **RCT:** randomised controlled trial; **RR:** risk ratio						
**GRADE Working Group grades of evidence** **High certainty:** we are very confident that the true effect lies close to that of the estimate of the effect. **Moderate certainty:** we are moderately confident in the effect estimate: the true effect is likely to be close to the estimate of the effect, but there is a possibility that it is substantially different. **Low certainty:** our confidence in the effect estimate is limited: the true effect may be substantially different from the estimate of the effect. **Very low certainty:** we have very little confidence in the effect estimate: the true effect is likely to be substantially different from the estimate of effect.						

^1^High risk of selection bias from inadequate allocation concealment (open list of random numbers). High risk of performance and detection bias as participants were aware of their group allocation. ^2^Participants who had marked improvement were compared to those who had little or no improvement. There is no comparison of those who had some improvement, compared to no improvement. ^3^Optimal information size was not reached (taken as < 400 participants for continuous outcomes or < 300 events for dichotomous outcomes, as a rule of thumb). ^4^High risk of performance and detection bias as participants were aware of their group allocation. High risk of attrition bias due to differential dropout between the two groups. ^5^Very wide confidence interval, which includes the possibility of potential harm and potential benefit from the intervention. ^6^Sample size extremely small (< 15 in each arm).

## Background

### Description of the condition

Vestibular migraine is a form of migraine in which a prominent symptom, often *the* predominant symptom, is recurrent attacks of vertigo (Dieterich 1999; Lempert 2009). These episodes of vertigo are associated with other migraine features, such as headache or sensitivity to light or sound. 

The diagnosis of vestibular migraine is challenging because of the overlap of some symptoms with both other balance disorders (such as Ménière's disease) and with headache migraine. People suffering from headache migraine may experience occasional vestibular symptoms, but this does not amount to a diagnosis of 'vestibular migraine'. 

There is now an agreed international classification system, which includes categories for 'definite' and 'probable' vestibular migraine (Lempert 2012; described in Appendix 1). In brief, a definite diagnosis of vestibular migraine requires at least five episodes of vestibular symptoms (of moderate to severe intensity) lasting between 5 minutes and 72 hours. At least half of the episodes must be associated with migrainous features (such as headache, photophobia, phonophobia or a visual aura) and individuals must also have a history of migraine. A diagnosis of 'probable' vestibular migraine requires similar features, but individuals have either migrainous features or a history of migraines (both are not required). Prior to this internationally agreed classification, the criteria proposed by Neuhauser and colleagues were widely used to identify people with vestibular migraine (Neuhauser 2001). There is a great deal of similarity between these classification systems, although the Neuhauser criteria do not require a certain number of episodes, or duration of episodes, to make the diagnosis. 

Vestibular migraine is the most common cause of recurrent spontaneous vertigo in adults (Dieterich 2016). The lifetime prevalence of vestibular migraine has been estimated at just under 1% (Neuhauser 2006) and, as such, it is much more common than Ménière's disease. A significant number of cases may still go undiagnosed because of unfamiliarity with the condition or the diagnostic criteria. The disorder may have a slight female preponderance (Lempert 2009). As with many migraine disorders, a genetic susceptibility has been described and candidate genes have been suggested (Frejo 2016).

The pathophysiology of vestibular migraine is still uncertain, but it seems likely to involve similar mechanisms to those of headache migraine. These include activation of the trigeminovascular system (TGVS), which receives nociceptive signals from the large intracranial vessels and the dura (Bernstein 2012). Activation of the TGVS results in neuronal stimulation within parts of the brain involved in pain perception and sensory processing (including the thalamus and the periaqueductal grey) and also causes the release of vasoactive neuropeptides, such as calcitonin gene‐related peptide (CGRP). These, in turn, cause dilatation of the meningeal vessels, extravasation of fluid from the vasculature and release of other inflammatory substances in the dura (Pietrobon 2003), creating a cycle of nerve stimulation. Cortical hyperexcitability, and subsequent cortical spreading depolarisation, also occurs. This may account for the aura or visual symptoms experienced by many migraineurs (Hadjikhani 2001). There may be overlap between headache migraine pathways and those of the vestibular system, accounting for the balance symptoms. For example, the trigeminovascular system receives pain signals from nerves of the dura mater and large intracranial blood vessels, but also from vessels of the inner ear (Vass 1998). Abnormal thalamic activation in response to vestibular stimulation has also been identified in patients with vestibular migraine (Russo 2014). CGRP itself is implicated in vestibular migraine, along with headache migraine, and increased CGRP levels have been linked to the development of symptoms in migraine (Villalón 2009). Work is ongoing into the relevance of CGRP in vestibular migraine, and whether pharmacological targeting of this molecule and its receptors will affect the condition.

The consequences of vestibular migraine for the individual may be considerable. The unpredictable, disabling attacks of vertigo or dizziness can be distressing and debilitating in equal measure. This has a considerable impact on engagement with day‐to‐day activities and overall quality of life. 

### Description of the intervention

Current pharmacological treatments for patients with vestibular migraine may be prophylactic, or used to treat an acute attack. Many are based on interventions that have been widely used to treat headache migraine. This review is focused on pharmacological interventions that are taken as prophylaxis, to prevent attacks occurring. 

A variety of pharmacological interventions have been used, or proposed, for prophylaxis of vestibular migraine symptoms. These include:

- beta‐blockers, for example propranolol;
- calcium channel blockers, including flunarizine;
- antiepileptics, such as sodium valproate, topiramate and gabapentin;
- antidepressants, including amitriptyline;
- diuretics, such as acetazolamide; 
- monoclonal antibodies against calcitonin gene‐related peptide (CGRP); 
- botulinum toxin;
- hormonal modification.

### How the intervention might work

There are currently two main targets for headache migraine prophylaxis ‐ either modulation of the pathways that process incoming nociceptive signals, or prevention of neuronal hyperexcitability (Ramadan 2007). These principles apply equally to the interventions used for vestibular migraine. 

Beta‐blockers have been used for many years in the prophylaxis of headache migraine. They may act by central blockade of β_1_ receptors, resulting in a reduction in norepinephrine release and synthesis. There may be additional effects on other neuronal pathways, for example through regulation of neuronal firing in the locus coeruleus and the periaqueductal grey (reviewed in Galletti 2009).  

The mechanism of action of calcium channel blockers in migraine is not fully understood. They may help to reduce cerebral hypoxia, by preventing vasoconstriction of central vessels, but other mechanisms of action have been suggested ‐ including effects on the nitric oxide pathways and serotonin (reviewed in Galletti 2009). 

Antiepileptics have multiple sites of action, but many increase GABA‐mediated neurotransmission, and block sodium and calcium channels, consequently reducing neuronal hyperexcitability (Vikelis 2010). 

Antidepressants are used for their effect on increasing serotonin levels. Tricyclic antidepressants (such as amitriptyline) and selective serotonin reuptake inhibitors act by preventing neuronal uptake of serotonin (and, for some drugs, norepinephrine), consequently increasing levels of these neurotransmitters (Galletti 2009).

Diuretics, in particular acetazolamide, have also been used in headache migraine (De Simone 2005), where the mechanism of action is postulated to be due to a direct effect on neuronal ion channels. 

CGRP is a neurotransmitter found in numerous locations within the central nervous system and peripheral sensory nerves. Levels of this neurotransmitter have been found to be elevated during headache migraine episodes (Goadsby 1990), and to decrease with the use of triptans (Goadsby 1993). Direct inhibition of the effect of CGRP using monoclonal antibodies may therefore have a potential therapeutic effect on migraine.

Botulinum toxin is increasingly used for relief of migraine headache, although the precise mechanism of action is still unclear (reviewed in Do 2018 and see also Herd 2018).

Headache migraine is well‐recognised to be influenced by hormonal fluctuations, with an increase in migraine frequency typically associated with menstruation and during the peri‐menopause, and an improvement in symptoms during pregnancy and post‐menopause (reviewed in Sacco 2012). Regulation of hormonal variation may therefore improve symptoms of migraine. However, care must be taken with the choice of treatment due to an increased risk of cardiovascular disease associated with combined oral contraceptives in sufferers of migraine with aura (Champaloux 2017).

### Why it is important to do this review

Balance disorders can be difficult to diagnose and treat. There are few specific diagnostic tests, a variety of related disorders, and a limited number of interventions that are known to be effective. To determine which topics within this area should be addressed with new or updated systematic reviews, we conducted a scoping and prioritisation process, involving stakeholders (https://ent.cochrane.org/balance-disorders-ent). Vestibular migraine was ranked as one of the highest priority topics during this process (along with persistent postural‐perceptual dizziness and Ménière's disease). 

The impact of vestibular migraine is considerable, with 40% of sufferers reporting sickness from work, and over 70% reporting the impact of their symptoms on daily activities as either moderate or severe (Neuhauser 2006). At present, there are no national or international guidelines to inform the management of this condition, therefore up‐to‐date, reliable evidence syntheses are required to help patients and healthcare professionals determine the benefits and harms of different interventions used for the condition. 

## Objectives

To assess the benefits and harms of pharmacological treatments used for prophylaxis of vestibular migraine.

## Methods

### Criteria for considering studies for this review

#### Types of studies

We included randomised controlled trials (RCTs) and quasi‐randomised trials (where trials were designed as RCTs, but the sequence generation for allocation of treatment used methods such as alternate allocation, birth dates etc). 

The number of episodes of vestibular migraine may vary with time ‐ patients sometimes have periods of more active disease, followed by a period of fewer attacks. Therefore cross‐over trials are not an appropriate study design when assessing prophylaxis for this condition. Cross‐over RCTs would only have been included if data could be extracted for the first phase of the study. If cluster‐RCTs were identified then they would have been eligible for inclusion, providing we could appropriately account for the clustering in the data analysis (according to methods described in the *Cochrane Handbook for Systematic Reviews of Interventions*) (Handbook 2021). However, we did not identify any cross‐over or cluster‐randomised trials for this review. 

#### Types of participants

We included studies that recruited participants with a diagnosis of vestibular migraine, according to the International Headache Society (IHS) and Bárány Society criteria (see Appendix 1). We also included studies that used other, established criteria, for example Neuhauser 2001. 

We included studies where participants were diagnosed with either 'definite' vestibular migraine or 'probable' vestibular migraine. 

Where studies recruited participants with a variety of diagnoses (e.g. vestibular migraine and headache migraine) we planned to include the study if either:

- the majority of participants (≥ 90%) had a diagnosis of vestibular migraine; or
- subgroup data were available that allowed us to identify data specifically from those with vestibular migraine. 

However, we did not identify any studies that included participants with headache migraine. 

#### Types of interventions

We included the following interventions:

- beta‐blockers;
- calcium channel blockers;
- antiepileptics;
- antidepressants;
- diuretics;
- monoclonal antibodies to CGRP or its receptor;
- botulinum toxin;
- hormonal modification.

The main comparisons were planned to be:

- beta‐blockers versus placebo/no treatment;
- calcium channel blockers versus placebo/no treatment;
- antiepileptics versus placebo/no treatment;
- antidepressants versus placebo/no treatment;
- diuretics versus placebo/no treatment;
- monoclonal antibodies to CGRP or its receptor versus placebo/no treatment;
- botulinum toxin versus placebo/no treatment;
- hormonal modification versus placebo/no treatment.

##### Concurrent treatments

There were no limits on the type of concurrent treatments used, providing these were used equally in each arm of the study. We planned to pool studies that included concurrent treatments with those where participants did not receive concurrent treatment, and to conduct subgroup analysis to determine whether the effect estimates may be different in those receiving additional treatment. 

#### Types of outcome measures

We assessed outcomes at the following time points:

- < 3 months;
- 3 to 6 months;
- > 6 to 12 months.

The exception was for adverse event data, when we used the longest time period of follow‐up. 

We searched the COMET database for existing core outcome sets of relevance to vestibular migraine and vertigo, but were unable to find any published core outcome sets. We therefore conducted a survey of individuals with experience of (or an interest in) balance disorders to help identify outcomes that should be prioritised. The results of this survey were used by the review author team to inform the choice of outcome measures in this review.

We analysed the following outcomes in the review (but we did not use them as a basis for including or excluding studies).

##### Primary outcomes

- Improvement in vertigo
  - Measured as a dichotomous outcome (improved/not improved), according to self‐report, or according to a change of a specified score (as described by the study authors) on a vertigo rating scale.
- Change in vertigo
  - Measured as a continuous outcome, to identify the extent of change in vertigo symptoms.
- Serious adverse events
  - Including any event that caused death, was life‐threatening, required hospitalisation, resulted in disability or permanent damage, or in congenital abnormality. Measured as the number of participants who experienced at least one serious adverse event during the follow‐up period.

Vertigo symptoms comprise a variety of different features, including frequency of episodes, duration of episodes and severity/intensity of the episodes. Where possible, we included data for the vertigo outcomes that encompassed all of these three aspects (frequency, duration and severity/intensity of symptoms). However, we anticipated that these data may not be available from all studies. If they were unavailable, then we extracted data on the frequency of vertigo episodes as an alternative measure for these outcomes. 

##### Secondary outcomes

- Disease‐specific health‐related quality of life
  - Measured with the Dizziness Handicap Inventory (DHI, Jacobsen 1990), a validated measurement scale in widespread use. If data from the DHI were unavailable we planned to extract data from alternative validated measurement scales, according to the order of preference described in the list below (based on the validity of the scales for this outcome):
    - DHI short form (Tesio 1999);
    - DHI screening tool (Jacobsen 1998).
  - Measured with tools to assess migraine‐related quality of life, such as the Migraine‐Specific Quality of Life Questionnaire (Jhingran 1998).
- Improvement in headache 
  - Measured as a dichotomous outcome (improved/not improved), according to self‐report, or according to a change of specified score (as described by the study authors) on a headache rating scale.
- Improvement in other migrainous symptoms 
  - Measured as a dichotomous outcome (improved/not improved), according to self‐report, or according to a change of specified score (as described by the study authors) on a rating scale.
  - Including nausea and vomiting, photophobia and phonophobia, visual aura. 
- Other adverse effects
  - Measured as the number of participants who experienced at least one episode of the specified adverse events during the follow‐up period. Including the following specified adverse effects:
    - gastrointestinal disturbance (e.g. nausea, vomiting, change in bowel habit);
    - sleep disturbance (drowsiness, tiredness or problems sleeping);
    - cardiovascular side effects (e.g. lightheadedness, palpitations);
    - numbness or paraesthesia;
    - dry mouth or blurred vision;
    - skin rash;
    - weight changes.

### Search methods for identification of studies

The Cochrane ENT Information Specialist conducted systematic searches for randomised controlled trials and controlled clinical trials. There were no language, publication year or publication status restrictions. The date of the search was 23 September 2022.

#### Electronic searches

The Information Specialist searched:

- the Cochrane ENT Trials Register (searched via the Cochrane Register of Studies to 23 September 2022);
- the Cochrane Central Register of Controlled Trials (CENTRAL) (searched via the Cochrane Register of Studies to 23 September 2022);
- Ovid MEDLINE(R) Epub Ahead of Print, In‐Process & Other Non‐Indexed Citations, Ovid MEDLINE(R) Daily and Ovid MEDLINE(R) (1946 to 23 September 2022);
- Ovid Embase (1974 to 23 September 2022);
- Web of Knowledge, Web of Science (1945 to 23 September 2022);
- ClinicalTrials.gov, www.clinicaltrials.gov (to 23 September 2022);
- World Health Organization (WHO) International Clinical Trials Registry Platform (ICTRP), https://trialsearch.who.int/ (to 23 September 2022).

The Information Specialist modelled subject strategies for databases on the search strategy designed for CENTRAL. The strategy has been designed to identify all relevant studies for a suite of reviews on various interventions for vestibular migraine (Webster 2022a; Webster 2022b; Webster 2022c). Where appropriate, they were combined with subject strategy adaptations of the highly sensitive search strategy designed by Cochrane for identifying randomised controlled trials and controlled clinical trials (as described in the Technical Supplement to Chapter 4 of the *Cochrane Handbook for Systematic Reviews of Interventions* version 6.1) (Lefebvre 2020). Search strategies for major databases including CENTRAL are provided in Appendix 2.

#### Searching other resources

We scanned the reference lists of identified publications for additional trials and contacted trial authors if necessary. In addition, the Information Specialist searched Ovid MEDLINE to retrieve existing systematic reviews relevant to this systematic review, so that we could scan their reference lists for additional trials. The Information Specialist also ran non‐systematic searches of Google Scholar to identify trials not published in mainstream journals.

We did not perform a separate search for adverse effects. We considered adverse effects described in included studies only.

### Data collection and analysis

#### Selection of studies

At least two review authors or co‐workers (of AD, KG, LHK, KW, SC) independently screened the remaining titles and abstracts using Covidence to identify studies that may be relevant for this review. Any discrepancies were resolved by consensus, or by retrieving the full text of the study for further assessment. 

We obtained the full text for any study that may have been relevant and two authors or co‐workers (of AD, KG, LHK, KW) again independently checked this to determine whether it met the inclusion criteria for the review. Any differences were resolved by discussion and consensus, or through recourse to a third author if necessary. 

We listed as excluded any studies that were retrieved in full text but subsequently deemed to be inappropriate for the review (according to the inclusion/exclusion criteria), according to the main reason for exclusion. 

The unit of interest for the review is the study, therefore multiple papers or reports of a single study have been grouped together under a single reference identification. We recorded the study selection process in sufficient detail to complete a PRISMA flow diagram (Figure 1) and the Characteristics of excluded studies table. 

**1:**
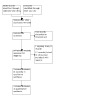
PRISMA flow chart of study retrieval and selection.

##### Screening eligible studies for trustworthiness

We assessed all studies meeting our inclusion criteria for trustworthiness using a screening tool developed by Cochrane Pregnancy and Childbirth. This tool includes specified criteria to identify studies that are considered sufficiently trustworthy to be included in the review (see Appendix 3). If any studies were assessed as being potentially 'high risk', we attempted to contact the study authors to obtain further information or address any concerns. We planned to exclude 'high risk' studies from the main analyses of the review if we were unable to contact the authors, or there was persisting uncertainty about the study, and only include studies with concerns as part of a sensitivity analysis (see Sensitivity analysis). The process is outlined in Figure 2.

**2:**
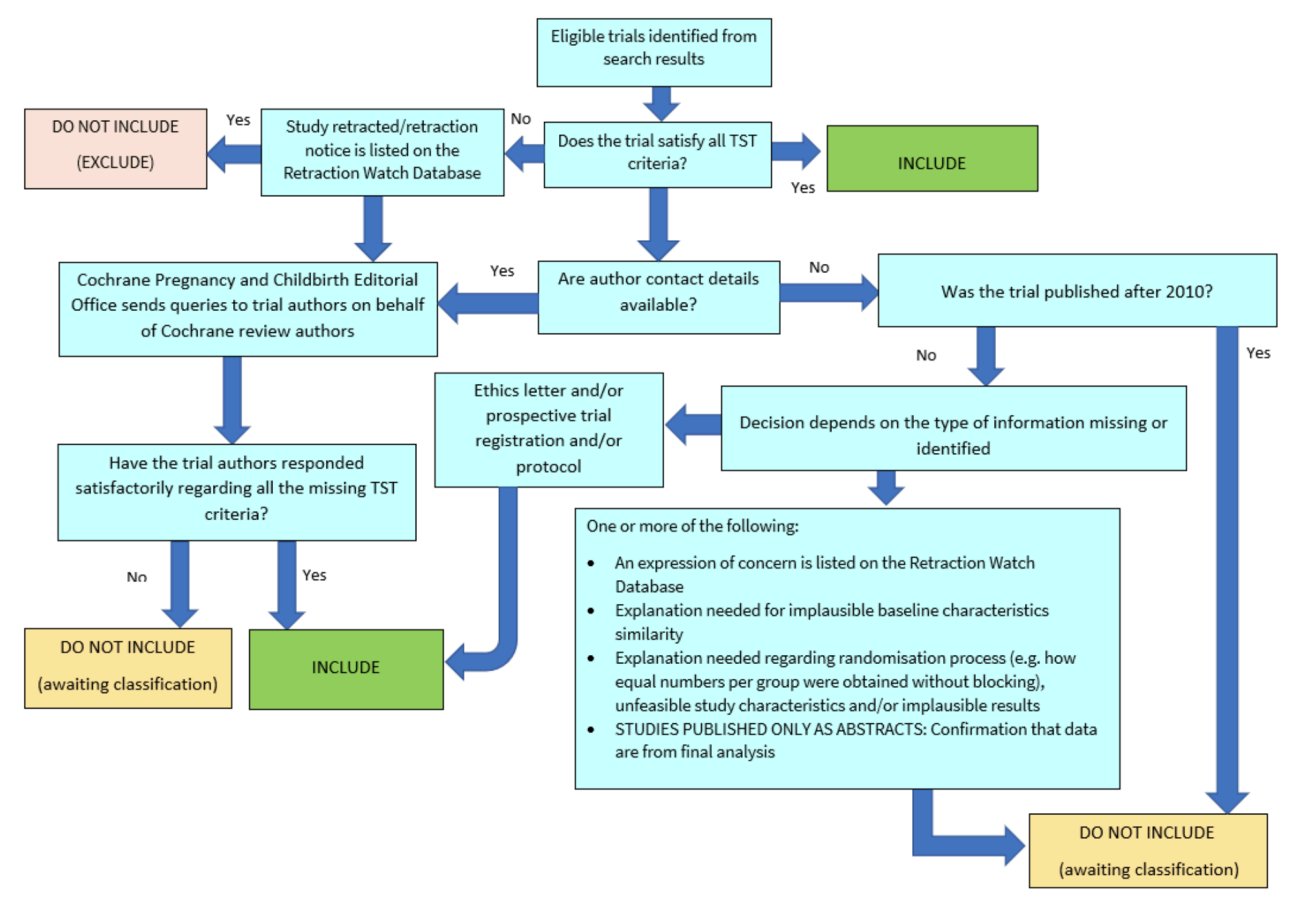
The Cochrane Pregnancy and Childbirth Trustworthiness Screening Tool

However, only one of the three studies included in this review satisfied all criteria for the screening tool (Bayer 2019). We noted that the study Lepcha 2014 was retrospectively registered, and no protocol was available. In addition, limited baseline characteristics were reported in the article, so we were unable to fully assess the two groups for differences and similarities. Similar concerns were identified with Yuan 2016, where no study protocol was identified, and baseline characteristics were reported for the entire cohort, not separately for the two groups. 

We attempted to contact study authors to clarify these issues, but received no reply. We had not anticipated this issue when drafting the protocol for our review, but it is likely to be a widespread issue for reviews that incorporate older studies.

There are several possible explanations for the studies that had concerns when using the tool. One is that there are issues with the trustworthiness of the studies identified in this review, and the data included may not give reliable estimates of the true effect. Alternatively, the trustworthiness screening tool may be excessively sensitive, and flag studies that are trustworthy, but where information has not been fully reported. We note that this tool (and others used for the same purpose) has not yet been validated for use. 

We therefore took the decision to include the studies in the review, despite the potential concerns over trustworthiness. The uncertainty in the results is captured as part of our GRADE rating in the certainty of the evidence, using the domain 'study limitations'. 

#### Data extraction and management

At least two review authors (of AD, LHK and KW) independently extracted outcome data from each study using a standardised data collection form. Where a study had more than one publication, we retrieved all publications to ensure complete extraction of data. Any discrepancies in the data extracted by the two authors were checked against the original reports, and differences were resolved through discussion and consensus, with recourse to a third author where necessary. If required, we contacted the study authors for clarification.

We included key characteristics of the studies, including the following information:

- study design, duration of the study, number of study centres and location, study setting and dates of the study;
- information on the participants, including the number randomised, those lost to follow‐up or withdrawn, the number analysed, the age of participants, gender, features of the condition (e.g. probable or definite vestibular migraine), diagnostic criteria used, inclusion and exclusion criteria for the individual studies;
- details of the intervention, comparator, and concomitant treatments or excluded medications;
- the outcomes specified and reported by the study authors, including the time points;
- funding for the study and any conflicts of interest for the study authors;
- information required to assess the risk of bias in the study, and to enable GRADE assessment of the evidence.

Once the extracted data had been checked and any discrepancies resolved, a single author transferred the information to Review Manager 5 (RevMan 2020). 

The primary effect of interest for this review is the effect of treatment assignment (which reflects the outcomes of treatment for people who were assigned to the intervention) rather than a per protocol analysis (the outcomes of treatment only for those who completed the full course of treatment as planned). For the outcomes of interest in this review, we extracted findings from the studies on an available case basis, i.e. all available data from all participants at each time point, based on the treatment to which they were randomised. This was irrespective of adherence, or whether participants had received the intervention as planned.

In addition to extracting pre‐specified information about study characteristics and aspects of methodology relevant to risk of bias, we extracted the following summary statistics for each study and outcome:

- For continuous data: the mean values, standard deviation and number of patients for each treatment group at the different time points for outcome measurement. Where change‐from‐baseline data were not available, we extracted the values for endpoint data instead. If values for the individual treatment groups were not reported, where possible we extracted summary statistics (e.g. mean difference) from the studies.
- For binary data: we extracted information on the number of participants experiencing an event, and the number of participants assessed at that time point. If values for the individual treatment groups were not reported, where possible we extracted summary statistics (e.g. risk ratio) from the studies.
- For ordinal scale data: if the data appear to be normally distributed, or if the analysis performed by the investigators indicated that parametric tests are appropriate, then we treated the outcome measure as continuous data. Alternatively, if data were available, we converted these to binary data for analysis.
- For time‐to‐event data: we did not identify any time‐to‐event data for this review. 

If necessary, we converted data found in the studies to a format appropriate for meta‐analysis, according to the methods described in the *Cochrane Handbook for Systematic Reviews of Interventions* (Handbook 2021). 

We pre‐specified time points of interest for the outcomes in this review. Where studies reported data at multiple time points, we took the longest available follow‐up point within each of the specific time frames. For example, if a study reported an outcome at 16 weeks and 20 weeks of follow‐up then the 20‐week data was included for the time point three to six months (12 to 24 weeks).

#### Assessment of risk of bias in included studies

Two authors (of AD, LHK, KW) undertook assessment of the risk of bias of the included studies independently, with the following taken into consideration, as guided by the *Cochrane Handbook for Systematic Reviews of Interventions* (Handbook 2011).

- sequence generation;
- allocation concealment;
- blinding;
- incomplete outcome data;
- selective outcome reporting; and
- other sources of bias.

We used the Cochrane risk of bias tool (Handbook 2011), which involves describing each of these domains as reported in the study and then assigning a judgement about the adequacy of each entry: 'low', 'high' or 'unclear' risk of bias.

#### Measures of treatment effect

We summarised the effects of dichotomous outcomes (e.g. serious adverse effects) as risk ratios (RR) with 95% confidence intervals (CIs). We have also expressed the results as absolute numbers based on the pooled results and compared to the assumed risk in the summary of findings tables (Table 1; Table 2) and full GRADE profiles (Table 3; Table 4). 

**1:** GRADE profile: Beta‐blockers versus placebo for prophylaxis of vestibular migraine

**Participants** **(studies)**	**Risk of bias**	**Inconsistency**	**Indirectness**	**Imprecision**	**Publication bias**	**Overall certainty of evidence**	**Study event rates (%)**		**Relative effect** **(95% CI)**	**Anticipated absolute effects**	
							**With placebo**	**With beta‐blockers**		**Risk with placebo**	**Risk difference with beta‐blockers**
**Change in vertigo frequency (follow‐up: range 3 months to 6 months; assessed with: number of vertigo attacks per month)**											
114 (1 RCT)	Serious^a^	Not serious	Not serious	Serious^b^	None	⨁⨁◯◯ Low	54	60	—	The mean vertigo frequency was **3.78** attacks per month	MD **0.3 attacks per month lower** (1.83 lower to 1.23 higher)
**Serious adverse events**											
121 (1 RCT)	Serious^a^	Not serious	Not serious	Very serious^b,c^	None	⨁◯◯◯ Very low	9/59 (15.3%)	6/62 (9.7%)	**RR 0.63** (0.24 to 1.67)	153 per 1000	**56 fewer per 1000** (from 116 fewer to 102 more)
**Disease‐specific health‐related quality of life (follow‐up: range 3 months to 6 months; assessed with: DHI mean score; scale from: 0 to 4, higher scores = worse quality of life)**											
90 (1 RCT)	Serious^a^	Not serious	Not serious	Serious^b^	None	⨁⨁◯◯ Low	41	49	—	The mean change in the DHI was an increase of **0.159** points over the follow‐up period	MD **0.08 points lower** (0.63 lower to 0.47 higher)
**Change in headache frequency (follow‐up: range 3 months to 6 months; assessed with: number of days with headache per month)**											
91 (1 RCT)	Serious^a^	Not serious	Not serious	Serious^b^	None	⨁⨁◯◯ Low	42	49	—	The mean headache frequency was **2.4** days with headache per month	MD **0.1 days with headache per month higher** (1.87 lower to 2.08 higher)

**CI:** confidence interval; **DHI:** Dizziness Handicap Inventory; **MD:** mean difference; **RCT:** randomised controlled trial; **RR:** risk ratio ^a^Risk of attrition bias due to substantial dropout over the course of the study. ^b^Optimal information size was not reached (taken as < 400 participants for continuous outcomes or < 300 events for dichotomous outcomes, as a rule of thumb). ^c^Wide confidence interval, which includes both the potential for considerable harm and potential benefit from the intervention.

**2:** GRADE profile: Calcium channel blockers versus no intervention for prophylaxis of vestibular migraine

**Participants** **(studies)** **Follow‐up**	**Risk of bias**	**Inconsistency**	**Indirectness**	**Imprecision**	**Publication bias**	**Overall certainty of evidence**	**Study event rates (%)**		**Relative effect** **(95% CI)**	**Anticipated absolute effects**	
							**With placebo**	**With calcium channel blockers**		**Risk with placebo**	**Risk difference with calcium channel blockers**
**Improvement in vertigo severity (follow‐up: range < 3 months; assessed with: "marked improvement" compared to "little/no improvement")**											
48 (1 RCT)	Very serious^a^	Not serious	Serious^b^	Serious^c^	None	⨁◯◯◯ Very low	14/23 (60.9%)	22/25 (88.0%)	**RR 1.45** (1.01 to 2.07)	609 per 1000	**274 more per 1000** (from 6 more to 651 more)
**Improvement in vertigo frequency (follow‐up: range 3 months to 6 months)**											
23 (1 RCT)	Very serious^d^	Not serious	Not serious	Very serious^c,e^	None	⨁◯◯◯ Very low	5/11 (45.5%)	9/12 (75.0%)	**RR 1.65** (0.80 to 3.41)	455 per 1000	**295 more per 1000** (from 91 fewer to 1000 more)
**Change in vertigo frequency (follow‐up: range 3 months to 6 months; assessed with: frequency of episodes over a 3‐month period)**											
23 (1 RCT)	Very serious^d^	Not serious	Not serious	Very serious^c,f^	None	⨁◯◯◯ Very low	11	12	—	The mean vertigo frequency was **5.5** episodes in 3 months	MD **3.3 episodes in 3 months lower** (5.94 lower to 0.66 lower)
**Improvement in headache (follow‐up: range < 3 months; assessed with: "marked improvement" compared to "little/no improvement")**											
48 (1 RCT)	Very serious^a^	Not serious	Serious^b^	Serious^c^	None	⨁◯◯◯ Very low	13/23 (56.5%)	19/25 (76.0%)	**RR 1.34** (0.88 to 2.05)	565 per 1000	**192 more per 1000** (from 68 fewer to 593 more)
**Other adverse effects ‐ drowsiness**											
71 (2 RCTs)	Very serious^g^	Not serious	Not serious	Very serious^c,e^	None	⨁◯◯◯ Very low	1/34 (2.9%)	5/37 (13.5%)	**Peto OR 3.85** (0.73 to 20.26)	29 per 1000	**75 more per 1000** (from 8 fewer to 351 more)
**Other adverse effects ‐ weight gain**											
71 (2 RCTs)	Very serious^g^	Not serious	Not serious	Very serious^c,e^	None	⨁◯◯◯ Very low	1/34 (2.9%)	3/37 (8.1%)	**Peto OR 2.58** (0.35 to 18.94)	29 per 1000	**43 more per 1000** (from 19 fewer to 335 more)

**CI:** confidence interval; **MD:** mean difference; **OR:** odds ratio; **RR:** risk ratio ^a^High risk of selection bias from inadequate allocation concealment (open list of random numbers). High risk of performance and detection bias as participants were aware of their group allocation. ^b^Participants who had marked improvement were compared to those who had little or no improvement. There is no comparison of those who had some improvement, compared to no improvement. ^c^Optimal information size was not reached (taken as < 400 participants for continuous outcomes or < 300 events for dichotomous outcomes, as a rule of thumb). ^d^High risk of performance and detection bias as participants were aware of their group allocation. High risk of attrition bias due to differential dropout between the two groups. ^e^Very wide confidence interval, which includes the possibility of potential harm and potential benefit from the intervention. ^f^Sample size extremely small (< 15 in each arm). ^g^Both studies had a high risk of performance and detection bias. Additional concerns were also present with both studies (attrition bias in Yuan 2016, selection bias in Lepcha 2014).

The reported event rate was zero for some outcomes. Therefore, we used the Peto odds ratio (OR) to analyse these data, according to the guidance in Xu 2021, as this should produce less biased estimates of the effect size when events are rare (as described in the Handbook 2021). 

For continuous outcomes, we expressed treatment effects as a mean difference (MD) with standard deviation (SD). We did not need to present any data using a standardised mean difference in this review. 

#### Unit of analysis issues

Vestibular migraine is unlikely to be a stable condition and interventions may not have a temporary effect. If cross‐over trials were identified then we planned to use only the data from the first phase of the study. If cluster‐randomised trials are identified then we will have ensured that analysis methods were used to account for clustering in the data according to the Handbook 2021. However, neither of these study designs were identified in the included studies. 

If we had identified studies with three or more arms, we would have ensured these were included to avoid double‐counting of any participants. However, this was not necessary for this review. 

#### Dealing with missing data

We tried to contact study authors via email whenever the outcome of interest is not reported, if the methods of the study suggested that the outcome had been measured. We planned to do the same if not all data required for meta‐analysis were reported (for example, standard deviations), unless we were able to calculate them from other data reported by the study authors. 

#### Assessment of heterogeneity

We assessed clinical heterogeneity by examining the included studies for potential differences between them in the types of participants recruited, interventions or controls used and the outcomes measured. 

We used the I^2^ statistic to quantify inconsistency among the studies in each analysis. We also considered the P value from the Chi^2^ test. If we had identified substantial heterogeneity, we planned to report this and explore possible causes through pre‐specified subgroup analysis, however this was not necessary. 

#### Assessment of reporting biases

We assessed reporting bias as within‐study outcome reporting bias and between‐study publication bias.

##### Outcome reporting bias (within‐study reporting bias)

We assessed within‐study reporting bias by comparing the outcomes reported in the published report against the study protocol or trial registry, whenever this could be obtained. If the protocol or trial registry entry was not available, we compared the outcomes reported to those listed in the methods section. If results were mentioned but not reported adequately in a way that allows analysis (e.g. the report only mentions whether the results were statistically significant or not), bias in a meta‐analysis is likely to occur. We planned to seek further information from the study authors in this situation. If no further information could be found, we noted this as being a 'high' risk of bias when the risk of bias tool was used. If there was insufficient information to judge the risk of bias we noted this as an 'unclear' risk of bias (Handbook 2011). 

##### Publication bias (between‐study reporting bias)

We planned to assess funnel plots if sufficient studies (more than 10) were available for an outcome. However, we did not identify sufficient studies to enable this. We did not identify any unpublished studies as part of this review. 

#### Data synthesis

##### Meta‐analysis of numerical data

Where possible and appropriate (if participants, interventions, comparisons and outcomes were sufficiently similar in the studies identified) we conducted a quantitative synthesis of results. We conducted all meta‐analyses using RevMan 2020. We anticipated that the underlying effect of the intervention may vary between studies, as there are likely to be differences between participants, settings and the interventions used for each study. We therefore planned to use a random‐effects method for meta‐analysis. However, as the only outcomes which we were able to meta‐analyse had few events, this necessitated the use of the fixed effect Peto odds ratio for analysis. We therefore explored whether the use of a random‐effects model substantially altered the effect estimates (see Sensitivity analysis). 

For dichotomous data, we analysed treatment differences as a risk ratio (RR) calculated using the Mantel‐Haenszel methods (or using a Peto odds ratio, as described above). We did not conduct any meta‐analysis for continuous data in this review. 

Improvement in vertigo symptoms may be assessed using a variety of methods, which consider different aspects of vertigo. These include:

- frequency of vertigo episodes;
- duration of vertigo episodes;
- severity/intensity of vertigo episodes;
- a composite measure of all of these aspects:
  - for example, assessed with a global score ‐ such as "how troublesome are your vertigo symptoms?", rated on an ordinal scale.

For the outcomes "improvement in vertigo" and "change in vertigo", we prioritised outcome measures that use a composite score ‐ encompassing aspects of vertigo frequency, duration and severity/intensity. Examples of this include a global rating scale of vertigo impact (rated from 0 to 10, where 0 is defined as no symptoms, and 10 is defined as the most troublesome symptoms) or the vertigo/balance subscale of the Vertigo Symptom Scale (Yardley 1992), or Vertigo Symptom Scale Short Form (Yardley 1998). Where data from composite scores were not available, we included data on the frequency of vertigo episodes as an alternative measure.

##### Synthesis using other methods

If we were unable to pool numerical data in a meta‐analysis for one or more outcomes we planned to provide a synthesis of the results using alternative methods, following the guidance in Chapter 12 of the Handbook 2021. However, this was not necessary, as results were typically provided by a single study. 

#### Subgroup analysis and investigation of heterogeneity

If statistical heterogeneity was identified for any comparisons, we planned to assess this considering the following subgroups:

- Different types of medication, within a specific class.
- Use of any concomitant treatment.
- Diagnosis of vestibular migraine
- Age of the participants
- Sex of the participants

However, due to the paucity of data available, and the few meta‐analyses included in this review, we did not carry out any subgroup analysis. 

#### Sensitivity analysis

We planned to carry out a number of sensitivity analyses for the primary outcomes in this review. However, the paucity of data and the lack of meta‐analyses has meant that this was not possible. 

If few studies are identified for meta‐analysis, the random‐effects model may provide an inaccurate measure of the between‐studies variance. Therefore, we planned to explore the impact of using a fixed‐effect model using a sensitivity analysis. However, few meta‐analyses were conducted, and these analyses were actually carried out using the Peto OR, a fixed‐effect method, due to zero events in at least one arm of a study. For completeness, we have compared the results to a random‐effects method using the Mantel‐Haenzel OR, but the results are very similar (Table 5).

**3:** Sensitivity analysis

**Analysis**	**Main analysis result**	**Method of sensitivity analysis**	**Sensitivity analysis result**
Analysis 2.5 Drowsiness	Peto OR 3.85 (95% CI 0.73 to 20.26)	Random‐effects, Mantel Haenszel OR	3.76 (95% CI 0.58 to 24.46)*
Analysis 2.5 Weight gain	Peto OR 2.58 (95% CI 0.35 to 18.94)	Random‐effects, Mantel Haenszel OR	2.25 (95% CI 0.31 to 16.26)*

CI: confidence interval; OR: odds ratio *Note that the primary analysis uses a Peto OR due to the occurrence of zero events in one arm of one study. Therefore, we have assessed the impact of changing to a random‐effects analysis using a Mantel‐Haenszel OR (as the Peto OR cannot use random‐effects).

We used the Cochrane Pregnancy and Childbirth Screening Tool to identify any studies with concerns over the data available. We had intended that any studies identified by the tool would be excluded from the main analyses in the review, but that we would explore the impact of including the data from these studies through a sensitivity analysis. However, as noted above, we had some concerns over the use of this tool, and few studies were included in the review, therefore this sensitivity analysis was not conducted. 

#### Summary of findings and assessment of the certainty of the evidence

Two independent authors (KG, KW) used the GRADE approach to rate the overall certainty of evidence using GRADEpro GDT (https://gradepro.org/) and the guidance in Chapter 14 of the *Cochrane Handbook for Systematic Reviews of Interventions* (Handbook 2021). Disagreements were resolved through discussion and consensus, or with recourse to a third author if necessary. The certainty of evidence reflects the extent to which we are confident that an estimate of effect is correct and we applied this in the interpretation of results. There are four possible ratings: high, moderate, low and very low. A rating of high certainty of evidence implies that we are confident in our estimate of effect and that further research is very unlikely to change our confidence in the estimate of effect. A rating of very low certainty implies that any estimate of effect obtained is very uncertain.

The GRADE approach rates evidence from RCTs that do not have serious limitations as high certainty. However, several factors can lead to the downgrading of the evidence to moderate, low or very low. The degree of downgrading is determined by the seriousness of these factors:

- Study limitations (risk of bias):
  - This was assessed using the rating from the Cochrane risk of bias tool for the study or studies included in the analysis. We rated down either one or two levels, depending on the number of domains that had been rated at high or unclear risk of bias. 
- Inconsistency:
  - This was assessed using the I^2^ statistic and the P value for heterogeneity for all meta‐analyses, as well as by visual inspection of the forest plot. For results based on a single study we rated this domain as no serious inconsistency.
- Indirectness of evidence:
  - We took into account whether there were concerns over the population included in the study or studies for each outcome, as well as whether additional treatments were offered that may impact on the efficacy of the intervention under consideration. 
- Imprecision:
  - We took into account the sample size and the width of the confidence interval for each outcome. If the sample size did not meet the optimal information size (i.e. < 400 people for continuous outcomes or < 300 events for dichotomous outcomes), or the confidence interval crossed the small effect threshold we rated down one level. If the sample size did not meet the optimal information size and the confidence interval included both potential harm and potential benefit we rated down twice. We also rated down twice for very tiny studies (e.g. 10 to 15 participants in each arm), regardless of the estimated confidence interval.
- Publication bias:
  - We considered whether there were likely to be unpublished studies that may impact on our confidence in the results obtained. 

We used a minimally contextualised approach, and rated the certainty in the interventions having an important effect (Zeng 2021). Where possible, we used agreed minimally important differences (MIDs) for continuous outcomes as the threshold for an important difference. Where no MID was identified, we provide an assumed MID based on agreement between the authors. For dichotomous outcomes, we looked at the absolute effects when rating imprecision, but also took into consideration the GRADE default approach (rating down when a RR crosses 1.25 or 0.80). We have justified all decisions to downgrade the certainty of the evidence using footnotes, and added comments to aid the interpretation of the findings, where necessary. 

We prepared a separate summary of findings table for the following comparisons:

- beta‐blockers versus placebo/no treatment;
- calcium channel blockers versus placebo/no treatment.

We included all primary outcomes in the summary of findings tables. We prioritised outcomes at the time point three to six months for presentation in the table. However, as some outcomes were only reported at earlier time points, these were also included. We have also included a full GRADE profile for all results and comparisons (Table 3; Table 4).

## Results

### Description of studies

#### Results of the search

The searches in September 2022 retrieved a total of 1186 records. This reduced to 558 after the removal of duplicates. We screened the titles and abstracts of the remaining 558 records. We discarded 534 records and assessed 24 full‐text records.

We excluded 14 studies (17 records) with reasons recorded in the review (see Excluded studies and Characteristics of excluded studies).

We identified one ongoing study, which is listed in Characteristics of ongoing studies.

We included three completed studies (six records) where results were available.

A flow chart of study retrieval and selection is provided in Figure 1.

#### Included studies

We included three RCTs (Bayer 2019; Lepcha 2014; Yuan 2016). Details of the individual studies can be found in the Characteristics of included studies.

##### Study design

All of the included studies were described as randomised controlled trials. They all included two arms, comparing an active medication to a placebo, or to no intervention. The duration of treatment ranged from three months (Lepcha 2014; Yuan 2016) to six months (Bayer 2019). The largest study was Bayer 2019, which recruited a total of 130 participants. 

##### Participants

All three studies recruited adult participants with a diagnosis of vestibular migraine. 

###### Diagnosis of vestibular migraine

The study Yuan 2016 appeared to use the IHS and Bárány Society criteria for the diagnosis of definite vestibular migraine (see Appendix 1 for details).

Bayer 2019 and Lepcha 2014 used the criteria proposed by Neuhauser 2001. The study Bayer 2019 included participants with either 'probable' (38%) or 'definite' (62%) vestibular migraine. It was not clear whether people with both definite and probable vestibular migraine were included in Lepcha 2014.

###### Features of vestibular migraine

None of the included studies gave details on the duration of the disease in trial participants. The authors of Bayer 2019 did state that participants in the trial had to experience between 6 and 30 attacks in the three‐month period preceding entry to the study. Similar criteria were applied in Yuan 2016, where participants needed to experience at least two attacks per month over the preceding three months. The attack frequency at baseline was not reported in Lepcha 2014. 

##### Interventions and comparisons

The studies evaluated two of our comparisons of interest. One evaluated metoprolol succinate (Bayer 2019) and the other two studies evaluated flunarizine (Lepcha 2014; Yuan 2016). 

###### Comparison 1: Beta‐blockers versus placebo

This comparison was evaluated by Bayer 2019. The authors used a dose of 95 mg oral metoprolol succinate once daily, for a period of six months. Short periods of up‐titration (one week of 47.5 mg daily) and tapering (two weeks of 47.5 mg daily) were also included at the start and end of the study.

###### Comparison 2: Calcium channel blockers versus no intervention

Both studies used a dose of 10 mg oral flunarizine once daily, to be taken at night. This was compared to no intervention in the control group. 

##### Outcomes

###### 1. Improvement in vertigo

For this outcome we included dichotomous data, assessed as the proportion of participants whose vertigo had 'improved' or 'not improved'. 

####### 1.1. Global score

The authors of Lepcha 2014 asked participants to rate the improvement in their vertigo symptoms during the study. This was conducted using a five‐point scale (0 to 4, with higher scores representing greater improvement), and analysed by comparing those who had little improvement (score 0 to 2) and those who had marked improvement (score 3 or 4). We considered it likely that this rating would encompass different aspects of vertigo (including the frequency, intensity and the duration of attacks) but this is not explicitly stated in the article. However, interpreting the results from the use of this scale is very challenging. For example, improvements in the frequency of vertigo may be offset by a corresponding increase in vertigo intensity, leading to little change on a global score. 

####### 1.2. Frequency

Yuan 2016 reported the proportion of participants in whom the frequency of vertigo episodes improved over the three‐month treatment period. 

###### 2. Change in vertigo

This outcome included data on the change in vertigo using a continuous numerical scale. 

####### 2.1. Global score

No studies reported the change in vertigo symptoms using a global score that considered the frequency, duration and intensity of vertigo attacks. 

####### 2.2. Frequency

Bayer 2019 reported on the monthly incidence rates of vertigo attacks at the end of the study. Yuan 2016 reported the frequency of vertigo episodes over the follow‐up period. 

###### 3. Serious adverse events

This outcome was assessed and reported by Bayer 2019. Yuan 2016 did not explicitly state that serious adverse events were assessed, although some other (non‐serious) adverse events are reported. 

###### 4. Disease‐specific health‐related quality of life

The authors of Bayer 2019 used the Dizziness Handicap Inventory to assess this outcome. 

###### 5. Improvement in headache

This was reported by Bayer 2019 as the mean number of days with headache per month, rather than as a dichotomous outcome. Improvement in headache was reported by Lepcha 2014 using the same score as used for vertigo. Again, we note the challenges in interpreting results from this global score. 

###### 6. Improvement in other migrainous symptoms

This outcome was not assessed by any of the included studies. 

###### 7. Other adverse effects

Other adverse effects were assessed by Bayer 2019, but they did not report specifically on the adverse events of interest in this review (gastrointestinal disturbance, sleep disturbance, cardiovascular side effects, numbness or paraesthesia, dry mouth or blurred vision, skin rash or weight changes). Sleep disturbance and weight gain were both assessed by Lepcha 2014 and Yuan 2016. 

#### Excluded studies

After assessing the full text, we excluded 14 studies (linked to 17 records) from this review. The main reason for exclusion for each study is listed below.

Three studies were not randomised controlled trials (ACTRN12616000683437; ChiCTR1800014766; CTRI/2019/09/021185).

Two articles were systematic reviews (Byun 2021; Wang 2020). We checked the reference lists of these to ensure that any relevant studies had been included in this review. 

One study recruited people with headache migraine, not vestibular migraine, therefore we excluded it due to the wrong population (Gordon 1993). 

Five studies used an incorrect comparator ‐ there was no placebo arm, or group that received no intervention, with which to compare the active intervention(s). These studies included:

- Liu 2017, which compared flunarizine, valproic acid and venlafaxine;
- Gode 2010, which compared high‐dose and low‐dose topiramate;
- Salviz 2015, which compared venlafaxine to propranolol;
- NCT05472675, which is an ongoing study comparing botulinum toxin and local anaesthetic to beta‐blockers;
- PACTR201909600414183, which compared topiramate to cinnarizine; and
- Staab 2015, which compared verapamil to sertraline.

We identified a trial registration for two planned RCTs that did appear to be relevant for this review (one comparing topiramate to placebo, the other comparing 4‐aminopyridine or atenolol to placebo). However, these studies were withdrawn prior to enrolment of any participants, therefore we excluded them (NCT00732108; NCT03578354).

### Risk of bias in included studies

See Figure 3 for the risk of bias graph (our judgements about each risk of bias item presented as percentages across all included studies) and Figure 4 for the risk of bias summary (our judgements about each risk of bias item for each included study). All the studies included had some concerns regarding the risk of bias, with at least two domains being rated at high risk of bias. 

**3:**
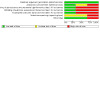
Risk of bias graph (our judgements about each risk of bias item presented as percentages across all included studies).

**4:**
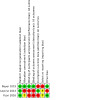
Risk of bias summary (our judgements about each risk of bias item for each included study).

#### Allocation

##### Random sequence generation

All three studies reported the use of a computerised randomisation schedule, therefore we rated them at low risk for this domain. 

##### Allocation concealment

The use of a web‐based system for randomisation and allocation meant that we rated the study Bayer 2019 at low risk of bias for allocation concealment. 

The trial registration for Lepcha 2014 stated that "an open list of random numbers" was used. We were concerned that this indicated that the randomisation schedule was freely available, and may therefore lead to a high risk of bias from inadequate concealment of allocation. 

There was no information available from Yuan 2016 regarding concealment of allocation, therefore we rated this domain at unclear risk of bias. 

#### Blinding

##### Blinding of participants and personnel

The authors of Bayer 2019 used a placebo‐controlled design to ensure that participants were unaware of their group allocation during the study. They also stated that study personnel were not aware of the group allocation for participants. We therefore rated this study at low risk of performance bias. 

Two studies were open‐label trials, with no placebo (the comparator group received no intervention), therefore participants and study personnel would have been aware of the group allocations (Lepcha 2014; Yuan 2016). We rated these studies at high risk of performance bias. 

##### Blinding of outcome assessors

In Bayer 2019, outcomes were reported by participants themselves using a daily diary. As participants were blinded to their treatment allocation, we rated this domain at low risk of bias.

As the studies by Lepcha 2014 and Yuan 2016 were open‐label, and outcome assessors were aware of the treatment allocation, we considered the risk of detection bias to be high. 

#### Incomplete outcome data

Bayer 2019 reported substantial dropout over the course of the study. This was sufficient to warrant a change in analysis plan from the protocol, to account for the missing data. Although this change may be valid and appropriate, we considered that the extent of missing data had the potential to have a large impact on the study results. Therefore, we rated this study at high risk of attrition bias. 

Yuan 2016 had imbalanced attrition between the two groups, with 22% loss to follow‐up in the control group, compared to 8% in the intervention group. We considered that this would be sufficient to impact on the results of the study, therefore we rated this domain at high risk of bias.  

There were few dropouts in Lepcha 2014, therefore we rated this study at low risk of attrition bias. 

#### Selective reporting

We rated Bayer 2019 at high risk of selective reporting bias. This was because of the change in analysis plan (as mentioned above, due to missing data) but also because outcomes were intended to be reported at nine months follow‐up (as well as six months) but no data were presented at this time point. 

The study Lepcha 2014 was registered with a clinical trial registry, but this was performed retrospectively. We were therefore unable to confirm whether the study had been conducted in accordance with a pre‐specified plan. We rated it at unclear risk of bias from selective reporting. We also rated Yuan 2016 at unclear risk, as no protocol was identified. 

#### Other potential sources of bias

We did not identify any other concerns over risk of bias for Bayer 2019 or Yuan 2016, therefore we rated this domain at low risk for these studies. 

We rated Lepcha 2014 at high risk of other bias. The method used to assess improvement in vertigo was unvalidated, and used an arbitrary cut‐off to distinguish people who experienced 'little improvement' as compared to 'marked improvement'. We were unable to determine the number of people who did not improve at all during the study. 

### Effects of interventions

See: Table 1; Table 2

#### 1. Beta‐blockers versus placebo 

A single study considered this comparison (Bayer 2019). 

##### 1.1. Improvement in vertigo

This outcome was not assessed. 

##### 1.2. Change in vertigo

###### 1.2.1. Frequency of vertigo

This outcome was assessed as the change in the frequency of vertigo episodes. No data were reported that considered a global score of vertigo severity. 

####### 1.2.1.1. < 3 months

No data were reported at this time point.

####### 1.2.1.2. 3 to 6 months

The mean difference in the frequency of episodes for those receiving metoprolol was a reduction of 0.30 episodes per month (95% confidence interval (CI) ‐1.83 to 1.23; 1 study; 114 participants; low‐certainty evidence; Analysis 1.1). The minimally important difference for a change in frequency of episodes has not been established, however we considered that a change of less than one episode per month may not be considered important by people with vestibular migraine. 

####### 1.2.1.3. > 6 to 12 months

No data were reported at this time point. 

##### 1.3. Serious adverse events

Bayer 2019 fully assessed and reported on serious adverse events during the study. The risk ratio (RR) for those receiving metoprolol was 0.63 but the confidence intervals were very wide (95% CI 0.24 to 1.67; 1 study; 121 participants; very low‐certainty evidence; Analysis 1.2).

##### 1.4. Disease‐specific health‐related quality of life

This was assessed and reported using the Dizziness Handicap Inventory (DHI). Due to the extent of missing data for individual questions on the DHI, the authors analysed this as the mean score per question. Therefore, the data are reported on a scale that ranges from 0 to 4 (higher scores representing worse quality of life), rather than the original DHI scale of 0 to 100. For ease of interpretation we have also provided the data transformed back onto the original scale. 

###### 1.4.1. < 3 months

No data were reported at this time point.

###### 1.4.2. 3 to 6 months

The mean difference in the DHI mean score at six months was ‐0.08 points (95% CI ‐0.63 to 0.47; 1 study; 91 participants; low‐certainty evidence; Analysis 1.3). If these data are transformed back to the original DHI score (range 0 to 100) then they would indicate a mean difference of ‐2 points (95% CI ‐15.75 to 11.75). This may be a trivial change, as the minimally important difference (MID) for the full DHI score has been suggested to be in the range of 11 to 18 points (Jacobsen 1990; Tamber 2009).

###### 1.4.3. > 6 to 12 months

No data were reported at this time point. 

##### 1.5. Improvement in headache

No dichotomous data were reported that assessed improvement in headache. However, the authors did report the change in headache frequency during the study. Due to the sparsity of other data we have included this as a surrogate measure for this outcome. 

###### 1.5.1. < 3 months

No data were reported at this time point.

###### 1.5.2. 3 to 6 months

The mean difference in the frequency of headaches per month for those receiving metoprolol was 0.10 (95% CI ‐1.87 to 2.08; 1 study; 91 participants; low‐certainty evidence; Analysis 1.4).

###### 1.5.3. > 6 to 12 months

No data were reported at this time point. 

##### 1.6. Improvement in other migrainous symptoms

This outcome was not assessed. 

##### 1.7. Other adverse effects

Data on adverse effects were collected by the trialists, but a breakdown of the individual adverse effects of interest in this review was not presented. 

#### 2. Calcium channel blockers versus no treatment

Two studies addressed this comparison (Lepcha 2014; Yuan 2016). 

##### 2.1. Improvement in vertigo

###### 2.1.2. Global score of vertigo

Lepcha 2014 assessed the improvement of vertigo using a global score. Participants were asked to rate the improvement in their vertigo symptoms using a five‐point scale: 0 = no improvement, 1 = mild improvement, 2 = moderate improvement, 3 = excellent improvement and 4 = completely asymptomatic. This five‐point scale was then separated into "little improvement" (scores 0 to 2) and "marked improvement" (scores 3 or 4). For the purpose of this analysis we were only able to compare those with marked improvement to those with little improvement, although we are aware that this may not accurately estimate the difference between those who experienced "no improvement" and "some improvement". 

####### 2.1.2.1. < 3 months

At 12 weeks, the risk ratio for improvement in those receiving flunarizine was 1.45 (95% CI 1.01 to 2.07; 1 study; 48 participants; very low‐certainty evidence; Analysis 2.1). 

####### 2.1.2.2. 3 to 6 months

No data were reported at this time point. 

####### 2.1.2.3. 6 to 12 months

No data were reported at this time point. 

###### 2.1.3. Frequency of vertigo

The authors of Yuan 2016 assessed the number of participants in whom the frequency of vertigo had improved over the course of the study. It was not clear how 'improvement' was defined ‐ we assume that this means a reduction in the number of vertigo episodes of at least one over the course of the study. 

####### 2.1.3.1. < 3 months

No data were reported at this time point.

####### 2.1.3.2. 3 to 6 months

At three months, the number of participants in whom the frequency of vertigo had improved was higher in the flunarizine group, with a risk ratio of 1.65, although the confidence interval was wide (95% 0.80 to 3.41; 1 study; 23 participants; very low‐certainty evidence; Analysis 2.2). 

####### 2.1.3.3. 6 to 12 months

No data were reported at this time point.  

##### 2.2. Change in vertigo

###### 2.2.1. Global score of vertigo

No data were reported that considered a global score of vertigo severity. 

###### 2.2.2. Frequency of vertigo

This outcome was assessed as the change in the frequency of vertigo episodes during a three‐month period. 

####### 2.2.2.1. < 3 months

No data were reported at this time point.

####### 2.2.2.2. 3 to 6 months

The mean difference in the frequency of episodes for those receiving flunarizine was a reduction of 3.30 episodes over a three‐month period (95% CI ‐5.94 to ‐0.66; 1 study; 23 participants; very low‐certainty evidence; Analysis 2.3). This would equate to a reduction of about one episode per month. The minimally important difference for a change in frequency of episodes has not been established, however we considered that a change of one episode per month may be considered important by people with vestibular migraine. 

####### 2.2.2.3. > 6 to 12 months

No data were reported at this time point. 

##### 2.3 Serious adverse events

It is not clear whether data on serious adverse events were specifically collected as part of these studies. The authors do report a small number of other adverse effects, which do not appear to be serious from the description (see below). We therefore presumed that there were no serious adverse events in either group, but this is not explicit. 

##### 2.4. Disease‐specific health‐related quality of life

This outcome was not assessed. 

##### 2.5. Improvement in headache

Lepcha 2014 assessed improvement in headache using the same score as for vertigo. Again, participants were grouped into those who had "little improvement" and those who had "marked improvement". 

###### 2.5.1. < 3 months

At 12 weeks, the risk ratio for improvement in headache for those receiving flunarizine was 1.34 (95% CI 0.88 to 2.05; 1 study; 48 participants; very low‐certainty evidence; Analysis 2.4). 

###### 2.5.2. 3 to 6 months

No data were reported at this time point. 

###### 2.5.3. 6 to 12 months

No data were reported at this time point. 

##### 2.6. Improvement in other migrainous symptoms

This outcome was not assessed. 

##### 2.7. Other adverse effects

The authors of both studies reported on some of our pre‐specified adverse effects of interest. As noted in Data synthesis, we used the Peto odds ratio for these analyses, due to the low event rates. The Peto odds ratio for drowsiness in those receiving flunarizine was 3.85 (95% CI 0.73 to 20.26; 2 studies; 71 participants; I^2^ = 0%; very low‐certainty evidence; Analysis 2.5). The Peto odds ratio for weight gain in those receiving flunarizine was 2.58 (95% CI 0.35 to 18.94; 2 studies; 71 participants; I^2^ = 0%; very low‐certainty evidence; Analysis 2.5). 

## Discussion

### Summary of main results

We identified only three studies for inclusion in this review. They evaluated two of our proposed interventions of interest: beta‐blockers and calcium channel blockers for prophylaxis of vestibular migraine. 

#### Beta‐blockers versus placebo

One study assessed this comparison ‐ participants were randomised to receive either 95 mg metoprolol once daily or a placebo. Metoprolol may make little or no difference to the frequency of vertigo episodes, disease‐specific quality of life and the frequency of headaches at between three and six months of follow‐up. The evidence regarding the occurrence of serious adverse effects was very uncertain. The other outcomes we prioritised in this review were not reported by the authors of this study (improvement in vertigo, improvement in other migrainous symptoms and other adverse effects). 

#### Calcium channel blockers versus no intervention

Two studies evaluated this comparison, both using a dose of 10 mg flunarizine daily. All of the evidence for this comparison was of very low certainty. Participants receiving flunarizine may be more likely to report improvement in vertigo (when assessed using a global score of vertigo symptoms), but the evidence is very uncertain. The frequency of vertigo episodes may also be slightly reduced for those who receive flunarizine but, again, the certainty of the evidence was very low. The evidence was very uncertain about the effect of flunarizine on improvement in vertigo (when assessed as a dichotomous outcome, rather than using a continuous scale) and headache. Flunarizine may result in an increase in the side effects of weight gain and drowsiness, but the confidence intervals were very wide and the evidence is very uncertain. We did not identify any data on serious adverse events, disease‐specific quality of life or other migrainous symptoms. 

### Overall completeness and applicability of evidence

We only identified three studies that were eligible for inclusion in this review, and they evaluated just two of the interventions of interest. No placebo‐controlled randomised controlled trials (RCTs) were identified that considered other interventions which have been used, or proposed for use, in people with vestibular migraine. This includes antiepileptics, antidepressants, diuretics, monoclonal antibodies to calcitonin gene‐related peptide (CGRP) or its receptor, botulinum toxin and hormonal treatment.

Two of the studies included in this review enrolled participants who were experiencing at least two attacks of vestibular migraine per month. Therefore, most of the evidence here may relate to individuals who have relatively frequent attacks, and may not apply to those who experience less frequent episodes. We note that vestibular and headache symptoms may vary considerably between different individuals, and may also fluctuate over time. In addition, follow‐up for all included studies was for between three and six months, so we do not have any evidence regarding longer‐term follow‐up. The limited data available also meant that we were unable to conduct any subgroup analysis, so the relative efficacy and harms of these interventions in different subgroups of people with vestibular migraine is uncertain. 

We did identify some evidence for most of our pre‐specified outcomes of interest in this review. However, no evidence was identified regarding associated migrainous symptoms (other than headache or vertigo), including photophobia, phonophobia, nausea and vomiting and visual aura. We also found only very limited information on potential adverse effects of these interventions. 

We noted that the description of outcome measures was sometimes inadequate in the studies ‐ it was difficult to identify how outcomes were assessed and whether this was appropriate. For example, the study Yuan 2016 reported on 'improvement' in vertigo frequency, but did not provide a clear description of how vertigo was judged to have improved. The study Lepcha 2014 considered improvement in vertigo using an ordinal scoring system, which did not appear to have been validated for use. In addition, a fairly arbitrary cut‐off was used to separate those who had 'marked improvement' from those who had 'little improvement'. The use of different tools for measuring symptoms of vestibular migraine makes it difficult to pool results across studies. In addition, many of these tools do not appear to have been subject to rigorous assessment and validation for measuring symptoms of vestibular migraine. Therefore it is difficult to know whether they accurately estimate the change in symptoms with treatment. 

### Quality of the evidence

We assessed all the evidence in this review as either low‐ or very low‐certainty, using the GRADE approach. This shows that our confidence in the estimates of effect is low, and that additional data from future studies are likely to change these estimates. 

Imprecision in the effect estimates was a major contributor to the low certainty of the evidence. The studies included in this review were all relatively small (ranging from 27 to 130 participants), and often the confidence intervals for any effect were wide, sometimes ranging from the possibility of a beneficial effect to the possibility of a harmful effect from the intervention.

We had concerns over the risk of bias for the studies included in the review. The studies Lepcha 2014 and Yuan 2016 were open‐label, where participants and study personnel were aware of the treatment allocation, leading to a high risk of performance bias and detection bias. There were also additional concerns in other domains for both of these studies. We considered the largest study at low risk of bias for most domains (Bayer 2019). However, due to considerable dropout during the trial we assessed it at high risk of attrition bias. However, we also had concerns over the possibility of selective reporting bias, as outcomes were not reported at the intended final follow‐up point.

As described above, we also had some concerns over the methods used to assess some outcomes. The certainty of the evidence was reduced for indirectness if the outcome had been assessed using an unvalidated scale, or the outcome reported did not fully align with our pre‐specified outcomes of interest. This was the case for improvement in vertigo and headache as reported by Lepcha 2014, where they compared participants with 'marked improvement' to those with 'little or no improvement', when we were intending to assess those with 'any improvement' compared to none. 

### Potential biases in the review process

A number of RCTs were excluded from this review as the comparator was incorrect ‐ an intervention was not compared to placebo or no treatment, but was instead compared to another (potentially) active intervention. This may be regarded as a source of bias in the review, although it is in accordance with our protocol. As the efficacy for different interventions in vestibular migraine is unknown, and there is no 'gold standard' treatment, we strongly felt that interventions must be compared to no treatment (or placebo treatment) in order to accurately estimate their effects. However, future reviews may consider addressing this problem with the use of network meta‐analysis. 

As noted in Selection of studies, we intended to use the Trustworthiness Screening Tool to select studies that would be included in the main analyses in this review. We had concerns regarding the methods used in two of the included studies when using this tool, but we were unable to establish contact with the authors to provide clarification. However, due to the paucity of data, and some concerns over the sensitivity of the tool, we decided to include all three studies in the main analyses of this review. Nonetheless, the evidence from these two studies is already rated as very low‐certainty, therefore the conclusions of this review are unlikely to be different, even if these studies were known to have problems in their conduct or reporting. 

### Agreements and disagreements with other studies or reviews

A previous Cochrane Review was prepared on this topic in 2016 but no studies were included (Maldonado Fernández 2015). At the time of publication for the previous review, the studies Bayer 2019 and Yuan 2016 had not been completed. The study Lepcha 2014 was also excluded from the original Cochrane Review, as the study used the Neuhauser 2001 criteria for diagnosis of vestibular migraine, rather than the International Headache Society (IHS) criteria. We considered that these two sets of diagnostic criteria were sufficiently similar that studies using either should be included in the review. 

We identified a recent systematic review that assessed the use of a number of different pharmacological interventions for vestibular migraine (antiepileptic drugs, calcium channel blockers, tricyclic antidepressants, beta‐blockers, serotonin and norepinephrine reuptake inhibitors), as well as vestibular rehabilitation (Byun 2021). This review differs from our own in two important ways. Firstly, the authors included non‐randomised studies as well as RCTs. In addition, meta‐analyses were conducted using data collected before and after treatment for all studies. Therefore, the comparison between randomised groups was not maintained (even when analysing data from RCTs) and the studies were analysed as if they were non‐comparative cohort studies. In this review, many of these treatments appeared to show efficacy for improvement in the frequency of vestibular migraine attacks, reduction (improvement) in Dizziness Handicap Inventory (DHI) scores and overall improvement in symptoms. However, there was no comparison with an appropriate control group for any of these analyses, therefore it is not possible to comment on how much of this improvement was related to the intervention itself. Given the fluctuation in symptoms of vestibular migraine we considered that these data may not accurately reflect the efficacy of these treatments. Nonetheless, Byun 2021 and colleagues do draw some similar conclusions to our own review, including the need for a longer duration of follow‐up, and the overall paucity of evidence from randomised controlled trials. 

Another recent systematic review evaluated the use of pharmacological (and non‐pharmacological) interventions for both the prophylaxis and acute treatment of vestibular migraine (Smyth 2022). Again, the authors of this review included both randomised and non‐randomised studies, therefore the results are not directly comparable with our own review. However, again the conclusions are similar ‐ that the overall evidence base for the treatment of vestibular migraine is of low certainty, and that well‐designed clinical trials are required in this area.  

## Authors' conclusions

**Implications for practice**

At present there are few placebo‐controlled randomised trials that evaluate pharmacological interventions for the prophylaxis of vestibular migraine. The only evidence we identified evaluated beta‐blockers (specifically, metoprolol) and calcium channel blockers (flunarizine), and we are aware that these medications may not be available in all countries. All of the evidence was of low or very low certainty, therefore we cannot be sure of the efficacy or potential harms of these interventions. People with vestibular migraine (and healthcare professionals who work with them) should be aware of this uncertainty in the evidence to help support decision‐making regarding the possible benefits and risks of treatment.

**Implications for research**

This review was conducted as part of a suite, which evaluate different interventions for the prophylaxis or acute treatment of vestibular migraine (Webster 2022a; Webster 2022b; Webster 2022c). The conclusions below relate to evidence from across the entire suite:

- There is a paucity of randomised controlled trials in this field, where active interventions are compared to no treatment or a placebo. Given the subjective nature of symptoms of vestibular migraine, the fluctuating severity of the condition and the lack of a 'gold standard' treatment, we consider that comparison with a placebo arm is vital to allow conclusions to be drawn on the efficacy and harms of different interventions. 
- Wherever possible, trialists should ensure that participants, study personnel and outcome assessors are appropriately blinded to the intervention, to reduce the risk of performance and detection bias affecting the results of studies. 
- Small, underpowered studies do little to improve the evidence base for these interventions. We would advocate the conduct of large, adequately powered, multicentre trials to ensure that more robust conclusions can be drawn from the study results. In addition, trialists need to be aware that there is considerable attrition over the course of these studies, and should be prepared to make additional efforts to improve follow‐up. 
- Future studies should also aim to follow up participants for longer periods of time, to identify whether interventions have lasting effects.
- There needs to be consensus on the appropriate outcomes to measure in trials that evaluate interventions for vestibular migraine, with input from different stakeholders, especially including those with the condition. As well as agreeing the types of outcomes that are important, the methods with which these are measured should be considered, including the use of validated scales (such as the Vestibular Migraine Patient Assessment Tool and Handicap Inventory (VM‐PATHI); Sharon 2020), to assess more subjective outcomes. This would be best achieved with the development of a core outcome set, analogous to that developed for use in trials of classical migraine (Haywood 2021). 

## History

Protocol first published: Issue 3, 2022

## Appendices

### Appendix 1. International Headache Society (IHS) and Bárány Society criteria for the diagnosis of vestibular migraine

From Lempert 2012:

**Vestibular migraine**

A. At least five episodes with vestibular symptoms of moderate or severe intensity, lasting five minutes to 72 hours.

B. Current or previous history of migraine with or without aura according to the International Classification of Headache Disorders (ICHD).

C. One or more migraine features with at least 50% of the vestibular episodes:

- headache with at least two of the following characteristics: one sided location, pulsating quality, moderate or severe pain intensity, aggravation by routine physical activity;
- photophobia and phonophobia;
- visual aura.

D. Not better accounted for by another vestibular or ICHD diagnosis.

**Probable vestibular migraine**

A. At least five episodes with vestibular symptoms of moderate or severe intensity, lasting five minutes to 72 hours.

B. Only one of the criteria B and C for vestibular migraine is fulfilled (migraine history *or *migraine features during the episode).

C. Not better accounted for by another vestibular or ICHD diagnosis.

To note: relevant vestibular symptoms are given as spontaneous vertigo, positional vertigo, visually induced vertigo, head motion‐induced vertigo or head motion‐induced dizziness with nausea. Moderate or severe symptoms are those that interfere with, and may prohibit, daily activities. 

### Appendix 2. Search strategies

The search strategies were designed to identify all relevant studies for a suite of reviews on various interventions for vestibular migraine.

**Table:**

**CENTRAL (CRS)**	**Cochrane ENT Register (CRS)**	**MEDLINE (Ovid)**
1 MeSH DESCRIPTOR Migraine Disorders Explode All AND CENTRAL:TARGET 2 MeSH DESCRIPTOR Vestibular Diseases AND CENTRAL:TARGET 3 MeSH DESCRIPTOR Vertigo AND CENTRAL:TARGET 4 MeSH DESCRIPTOR Dizziness Explode All AND CENTRAL:TARGET 5 #2 OR #3 OR #4 AND CENTRAL:TARGET 6 #1 AND #5 AND CENTRAL:TARGET 7 (migrain* adj5 (vertig* or dizz* or vestibul* or spinning)):AB,EH,KW,KY,MC,MH,TI,TO AND CENTRAL:TARGET 8 #7 OR #6 AND CENTRAL:TARGET	1 MeSH DESCRIPTOR Migraine Disorders Explode All AND INREGISTER 2 MeSH DESCRIPTOR Vestibular Diseases AND INREGISTER 3 MeSH DESCRIPTOR Vertigo AND INREGISTER 4 MeSH DESCRIPTOR Dizziness Explode All AND INREGISTER 5 #2 OR #3 OR #4 AND INREGISTER 6 #1 AND #5 AND INREGISTER 7 (migrain* adj5 (vertig* or dizz* or vestibul* or spinning)):AB,EH,KW,KY,MC,MH,TI,TO AND INREGISTER 8 #7 OR #6 AND INREGISTER 9 * AND CENTRAL:TARGET 10 #8 NOT #9	1 exp Migraine Disorders/ 2 Vestibular Diseases/ 3 Vertigo/ 4 exp Dizziness/ 5 2 or 3 or 4 6 1 and 5 7 (migrain* adj5 (vertig* or dizz* or vestibul* or spinning)).ab,ti. 8 6 or 7 9 randomized controlled trial.pt. 10 controlled clinical trial.pt. 11 randomized.ab. 12 placebo.ab. 13 drug therapy.fs. 14 randomly.ab. 15 trial.ab. 16 groups.ab. 17 9 or 10 or 11 or 12 or 13 or 14 or 15 or 16 18 exp animals/ not humans.sh. 19 17 not 18 20 8 and 19
**Embase (Ovid)**	**Web of Science Core Collection (Web of Knowledge)**	**Trial Registries**
1. exp vestibular migraine/ 2. (migrain* adj5 (vertig* or dizz* or vestibul* or spinning)).ab,ti. 3. 1 or 2 4. Randomized controlled trial/ 5. Controlled clinical study/ 6. Random$.ti,ab. 7. randomization/ 8. intermethod comparison/ 9. placebo.ti,ab. 10. (compare or compared or comparison).ti. 11. ((evaluated or evaluate or evaluating or assessed or assess) and (compare or compared or comparing or comparison)).ab. 12. (open adj label).ti,ab. 13. ((double or single or doubly or singly) adj (blind or blinded or blindly)).ti,ab. 14. double blind procedure/ 15. parallel group$1.ti,ab. 16. (crossover or cross over).ti,ab. 17. ((assign$ or match or matched or allocation) adj5 (alternate or group$1 or intervention$1 or patient$1 or subject$1 or participant$1)).ti,ab. 18. (assigned or allocated).ti,ab. 19. (controlled adj7 (study or design or trial)).ti,ab. 20. (volunteer or volunteers).ti,ab. 21. human experiment/ 22. trial.ti. 23. 4 or 5 or 6 or 7 or 8 or 9 or 10 or 11 or 12 or 13 or 14 or 15 or 16 or 17 or 18 or 19 or 20 or 21 or 22 24. (random$ adj sampl$ adj7 ("cross section$" or questionnaire$1 or survey$ or database$1)).ti,ab. 25. comparative study/ or controlled study/ 26. randomi?ed controlled.ti,ab. 27. randomly assigned.ti,ab. 28. 25 or 26 or 27 29. 24 not 28 30. Cross‐sectional study/ 31. randomized controlled trial/ or controlled clinical study/ or controlled study/ 32. (randomi?ed controlled or control group$1).ti,ab. 33. 31 or 32 34. 30 not 33 35. (((case adj control$) and random$) not randomi?ed controlled).ti,ab. 36. (Systematic review not (trial or study)).ti. 37. (nonrandom$ not random$).ti,ab. 38. "Random field$".ti,ab. 39. (random cluster adj3 sampl$).ti,ab. 40. (review.ab. and review.pt.) not trial.ti. 41. "we searched".ab. 42. review.ti. or review.pt. 43. 41 and 42 44. "update review".ab. 45. (databases adj4 searched).ab. 46. (rat or rats or mouse or mice or swine or porcine or murine or sheep or lambs or pigs or piglets or rabbit or rabbits or cat or cats or dog or dogs or cattle or bovine or monkey or monkeys or trout or marmoset$1).ti. and animal experiment/ 47. 29 or 34 or 35 or 36 or 37 or 38 or 39 or 40 or 43 or 44 or 45 48. 23 not 47 49. 3 and 48	# 3 #2 AND #1 Indexes=SCI‐EXPANDED, CPCI‐S Timespan=All years # 2 TOPIC: (((randomised OR randomized OR randomisation OR randomisation OR placebo* OR (random* AND (allocat* OR assign*) ) OR (blind* AND (single OR double OR treble OR triple) )))) Indexes=SCI‐EXPANDED, CPCI‐S Timespan=All years # 1 TOPIC: (migrain* NEAR/5 (vertig* or dizz* or vestibul* or spinning) ) Indexes=SCI‐EXPANDED, CPCI‐S Timespan=All years	**Clinicaltrials.gov** ( migraine OR migrainous ) AND ( vertigo OR dizziness OR dizzy OR vertiginous OR vestibular OR spinning ) **ICTRP** migrain* AND (vertig* OR dizz* OR vestibul* OR spinning)

### Appendix 3. Trustworthiness Screening Tool

This screening tool has been developed by Cochrane Pregnancy and Childbirth. It includes a set of predefined criteria to select studies that, based on available information, are deemed to be sufficiently trustworthy to be included in the analysis. These criteria are:

**Research governance**

- Are there any retraction notices or expressions of concern listed on the Retraction Watch Database relating to this study?
- Was the study prospectively registered (for those studies published after 2010)? If not, was there a plausible reason?
- When requested, did the trial authors provide/share the protocol and/or ethics approval letter?
- Did the trial authors engage in communication with the Cochrane Review authors within the agreed timelines?
- Did the trial authors provide IPD data upon request? If not, was there a plausible reason?

**Baseline characteristics**

- Is the study free from characteristics of the study participants that appear too similar (e.g. distribution of the mean (SD) excessively narrow or excessively wide, as noted by Carlisle 2017)?

**Feasibility**

- Is the study free from characteristics that could be implausible? (e.g. large numbers of women with a rare condition (such as severe cholestasis in pregnancy) recruited within 12 months);
- In cases with (close to) zero losses to follow‐up, is there a plausible explanation?

**Results**

- Is the study free from results that could be implausible? (e.g. massive risk reduction for main outcomes with small sample size)?
- Do the numbers randomised to each group suggest that adequate randomisation methods were used (e.g. is the study free from issues such as unexpectedly even numbers of women ‘randomised’ including a mismatch between the numbers and the methods, if the authors say ‘no blocking was used’ but still end up with equal numbers, or if the authors say they used ‘blocks of 4’ but the final numbers differ by 6)?

Studies assessed as being potentially ‘high risk’ will be not be included in the review. Where a study is classified as ‘high risk’ for one or more of the above criteria we will attempt to contact the study authors to address any possible lack of information/concerns. If adequate information remains unavailable, the study will remain in ‘awaiting classification’ and the reasons and communications with the author (or lack of) described in detail.

The process is described in full in Figure 2. 

## Data and analyses

**Comparison 1:** Beta‐blockers versus placebo

Outcome or subgroup title	No. of studies	No. of participants	Statistical method	Effect size
1.1 Change in vertigo frequency	1	114	Mean Difference (IV, Random, 95% CI)	‐0.30 [‐1.83, 1.23]
1.2 Serious adverse events	1	121	Risk Ratio (M‐H, Random, 95% CI)	0.63 [0.24, 1.67]
1.3 Disease‐specific health‐related quality of life	1	90	Mean Difference (IV, Random, 95% CI)	‐0.08 [‐0.63, 0.47]
1.4 Change in headache frequency	1	91	Mean Difference (IV, Random, 95% CI)	0.10 [‐1.87, 2.08]

**Comparison 2:** Calcium channel blockers versus no intervention

Outcome or subgroup title	No. of studies	No. of participants	Statistical method	Effect size
2.1 Improvement in vertigo severity	1	48	Risk Ratio (M‐H, Random, 95% CI)	1.45 [1.01, 2.07]
2.2 Improvement in vertigo frequency	1	23	Risk Ratio (M‐H, Random, 95% CI)	1.65 [0.80, 3.41]
2.3 Change in vertigo frequency	1	23	Mean Difference (IV, Random, 95% CI)	‐3.30 [‐5.94, ‐0.66]
2.4 Improvement in headache	1	48	Risk Ratio (M‐H, Random, 95% CI)	1.34 [0.88, 2.05]
2.5 Other adverse effects	2		Peto Odds Ratio (Peto, Fixed, 95% CI)	Subtotals only
2.5.1 Drowsiness	2	71	Peto Odds Ratio (Peto, Fixed, 95% CI)	3.85 [0.73, 20.26]
2.5.2 Weight gain	2	71	Peto Odds Ratio (Peto, Fixed, 95% CI)	2.58 [0.35, 18.94]

## Characteristics of studies

### Characteristics of included studies [ordered by study ID]

**Table:** Bayer 2019

***Study characteristics***		
Methods	Multicentre, parallel‐group, double‐blind, placebo‐controlled RCT with 6 months duration of treatment and follow‐up	
Participants	**Setting: **the trial was conducted at 6 sites in Germany, with participants recruited from academic outpatient clinics, and treated as outpatients. Recruitment took place between 20 June 2012 and 10 April 2017. **Sample size:** **Number randomised:** 130 participants **Number completed:** 91 participants **Participant baseline characteristics** **Age:** Metoprolol succinate group: 44.4 (SD 14.2) years Placebo group: 42.8 (SD 14.3) years **Gender:** Metoprolol succinate group**:** 22 males (33.8%); 43 females (66.2%) placebo: 29 males (44.6%); 36 females (55.4%) **Probable/definite vestibular migraine: ** Metoprolol succinate group: Probable VM n = 23 (35.4%) Definite VM n = 42 (64.6%) Placebo group: Probable VM n = 27 (41.5%) Definite VM n = 38 (58.5%) ** Attack frequency at baseline: ** Not reported; inclusion criterion was between 6 and 30 attacks in a 3‐month period **Duration of disease: ** Not reported **Inclusion criteria:** People with a diagnosis of probable or definite vestibular migraine according to the criteria of Neuhauser 2001: Episodic vestibular symptoms of at least moderate severity (rotational vertigo, other illusory self or object motion, positional vertigo, head motion intolerance, i.e. sensation of imbalance or illusory self or object motion that is provoked by head motion) Migraine according to the International Headache Society (IHS) criteria At least one of the following migrainous symptoms during at least 2 vertiginous attacks: migrainous headache, photophobia, phonophobia, visual or other auras Other causes ruled out by appropriate investigations A frequency of between 6 and 30 vestibular migraine attacks in the 3 months prior to the screening visit Aged 18 to 80 years **Exclusion criteria:** Other vestibular disorders such as Ménière's disease, phobic postural vertigo, benign paroxysmal positioning vertigo, vestibular paroxysmia. Central disorders such as paroxysmal brainstem attacks or transient ischaemic attacks (TIAs). Contraindications for the treatment with metoprolol (e.g. allergic reaction to one of the trial drugs, pregnancy or breastfeeding, sinoatrial (SA)‐block, atrioventricular (AV)‐block, sick sinus syndrome, bradycardia less than 50 bpm at rest, systolic blood pressure less than 100 mmHg, end‐grade peripheral arterial disease and bronchial asthma). Other medical conditions, including phaeochromocytoma, poorly controlled diabetes mellitus, porphyria, psoriasis, disorders of haemostasis, severe coronary heart disease or heart failure. Certain concurrent medications, such as monoamine oxidase (MAO)‐inhibitors or sympathomimetic drugs. Persistent hypertension with systolic blood pressure greater than 180 mmHg or diastolic BP greater than 110 mmHg (mean of 3 consecutive arm‐cuff readings over 20 to 30 minutes) that cannot be controlled by anti‐hypertensive therapy. Life expectancy less than 12 months or other serious illness, e.g. severe hepatic, cardiac or renal failure, acute myocardial infarction, neoplasm or a complex disease that may confound treatment assessment. Participation in another study with an investigational drug or device within the last 30 days, prior participation in the current study, or planned participation in another trial. **Diagnosis of vestibular migraine:** The criteria proposed by Neuhauser 2001 were used. These include a diagnosis of probable (criteria 1, 4 and 5) or definite (criteria 1, 2, 3 and 5) vestibular migraine: Episodic vestibular symptoms of at least moderate severity (rotational vertigo, other illusory self or object motion, positional vertigo, head motion intolerance, i.e. sensation of imbalance or illusory self or object motion that is provoked by head motion) Migraine according to the IHS criteria At least one of the following migrainous symptoms during at least 2 vertiginous attacks: migrainous headache, photophobia, phonophobia, visual or other auras At least one of the following: migraine according to the IHS criteria; migrainous symptoms during vertigo (as specified in 3); migraine‐specific precipitants of vertigo, e. g. specific foods, sleep irregularities, hormonal changes; response to anti‐migraine drugs Other causes ruled out by appropriate investigations	
Interventions	**Intervention (n = 65 randomised, n = 49 completed):** metoprolol succinate. The treatment comprised a 1‐week run‐in period of 47.5 mg metoprolol succinate per day. This was followed by a 6‐month maintenance treatment period with 95 mg once daily, and finally a tapering dose at the end of the study (47.5 mg once daily for 2 weeks before stopping completely). **Comparator (n = 65 randomised, n = 42 completed):** placebo tablets were identical in appearance to the study medication and were taken in the same way **Background interventions administered to all participants ** No medications were used as standard in this trial Acute medical treatment of VM‐related attacks was allowed, such as with non‐opioid analgesics, NSAIDs or triptans. If the participant was on prophylactic treatment for migraine, a washout period of at least 1 month was required before enrolment. Topiramate, valproic acid, lamotrigine, tricyclic antidepressants and other beta‐blockers were considered as prohibited concomitant medication and thus a protocol violation.	
Outcomes	**Primary outcomes relevant to this review:** **Improvement in vertigo** This outcome was not reported **Change in vertigo** This was reported as vertigo frequency, with the monthly incidence rates of vertigo attacks **Serious adverse events** Serious adverse events were systematically assessed and reported **Secondary outcomes relevant to this review:** **Disease‐specific health‐related quality of life** Assessed with the DHI. Results are reported as the mean score for each item (rather than a total score) to account for missing data. Range is therefore 0 to 4 points in original publication (rather than full score which has a range of 0 to 100). **Improvement in headache** This was not reported as a dichotomous outcome. Instead, the authors reported the mean number of monthly headache days at months 4 to 6. **Improvement in other migrainous symptoms ** Not reported **Other adverse effects** Adverse events were systematically assessed and reported, but there was no report of the adverse events that we prioritised for this review **Other outcomes reported in the study:** Smooth pursuit eye movement Subjective visual vertical	
Funding sources	Quote: "The PROVEMIG study was not co‐sponsored and was supported by the German Center for Vertigo and Balance Disorders (DSGZ), University Hospital Munich, Campus Grosshaden, funded by the German Federal Ministry of Education and Research within the framework of the Integrated Research and Treatment Centers program (funding reference number: 01EO0901). The funder had no role in the design, management, data collection, analyses, or interpretation of the data or in writing the manuscript or the decision to submit for publication."	
Declarations of interest	Quote: "MS [Michael Strupp] is Joint Chief Editor of the Journal of Neurology, Editor in Chief of Frontiers of Neuro‐otology and Section Editor of F1000. He has received speaker’s honoraria from Abbott, Actelion, Auris Medical, Biogen, Eisai, Grünenthal, GSK, Henning Pharma, Interacoustics, MSD Sharp & Dohme, Otometrics, Pierre‐Fabre, TEVA GmBH, and UCB. He is a shareholder in IntraBio. He acts as a consultant for Abbott, Actelion, AurisMedical, Heel, IntraBio and Sensorion. The remaining authors declare that they have no competing interests."	
Notes	**Research integrity checklist:** No retractions or expressions of concern The trial was prospectively registered No concerns over baseline characteristics of participants Plausible loss to follow‐up was reported No implausible results are reported Blocked randomisation was used, which accounts for similar numbers allocated to each group	
***Risk of bias***		
**Bias**	**Authors' judgement**	**Support for judgement**
Random sequence generation (selection bias)	Low risk	Quote from study protocol: "The randomization technique is based on randomized balanced blocks with random block length. The procedure considers stratification by the center", "an internet‐based randomization tool ("Randoulette"), which chooses one of the trial kits stored at the respective center when a new patient fulfills the inclusion criteria and has signed the informed consent." From article: "The concealed allocation was performed by an internet‐based randomization schedule stratified by study site (https://wwwapp.ibe.med.uni‐muenchen.de/randoulette). The fixed block size was four (starting with 6) which was not disclosed during the trial. The random number list was generated by a person with no clinical involvement in the trial." Comment: computer‐based randomisation method
Allocation concealment (selection bias)	Low risk	Quote from protocol: "neither the investigators nor the patients will be informed about the treatment arm to which a patient is allocated. Neither can get access to the randomization list." "The concealed allocation was performed by an internet‐based randomization schedule stratified by study site (https://wwwapp.ibe.med.uni‐muenchen.de/randoulette)."
Blinding of participants and personnel (performance bias) All outcomes	Low risk	Quote: "Metoprolol [...] tablets [...] were encapsulated for blinding purposes. Hard gelatine capsules containing the active ingredient were refilled from original pharmacy packaging into re‐labelled blisters [...]. Placebo was an identically appearing inactive capsule filled with mannitol and aerosil that did not contain any active ingredient; this was packed in blisters that looked identical to those of the investigational drug" Comment: adequate blinding of intervention. Patients and site personnel including outcome assessors, data analysts and statisticians remained blinded to treatment allocation.
Blinding of outcome assessment (detection bias) All outcomes	Low risk	Comment: outcomes were subjectively reported by blinded participants.
Incomplete outcome data (attrition bias) All outcomes	High risk	Comment: substantial dropout over the course of the trial, and missing data required a change in analysis methods (to allow for imputation to account for missing data).
Selective reporting (reporting bias)	High risk	Comment: although there are good reasons for the methods used, the analysis methods for the primary outcome were changed from the protocol. The protocol also states that some outcomes will be reported at 9 months (after discontinuation of treatment), but no data are presented for this time point ‐ potentially because of the inconclusive findings from the treatment period.
Other bias	Low risk	Comment: no other concerns were noted.

**Table:** Lepcha 2014

***Study characteristics***		
Methods	Two‐arm, parallel‐group, open‐label RCT with 12 weeks duration of treatment and follow‐up	
Participants	**Setting: **single‐centre study, conducted from the audiovestibular clinic at a tertiary care referral hospital in India. Participants were enrolled between July 2010 and August 2011. **Sample size:** **Number randomised:** 52 participants **Number completed:** 48 participants **Participant baseline characteristics** **Age: ** Flunarizine group: < 24 years: 6 (23%) 25 to 34 years: 14 (54%) > 35 years: 6 (23%) Control group: < 24 years: 6 (23%) 25 to 34 years: 9 (34.7%) > 35 years: 11 (42.3%) **Gender: ** Flunarizine group: 10 males (38.5 %), 16 females (61.5%) Control group: 8 males (30.7%), 18 females (69.3%) **Probable/definite vestibular migraine: ** Not reported **Attack frequency at baseline: ** Not reported **Duration of disease: ** Not reported **Inclusion criteria:** Aged 18 to 75. Diagnosis of migrainous vertigo according to Neuhauser 2001 criteria. Both male and female participants were recruited. **Exclusion criteria:** People with associated benign paroxysmal positional vertigo, Ménière’s disease, chronic discharging ear, past history of ear surgery, profound hearing loss, stroke, intracranial tumours or those on calcium channel blockers for hypertension were excluded from the study **Diagnosis of vestibular migraine:** The Neuhauser 2001 criteria were used. The authors do not state whether those with probable and/or definite vestibular migraine were included.	
Interventions	**Intervention (n = 26 randomised, n = 25 completed):** flunarizine 10 mg once daily at bedtime **Comparator (n = 26 randomised, n = 23 completed):** no intervention **Background interventions administered to all participants:** betahistine (16 mg) was given 3 times daily for 48 hours at the onset of a vertiginous attack, and paracetamol (1 g) was taken for acute attacks of headache for both arms. Both groups were instructed to carry out active vestibular exercises**. **	
Outcomes	**Primary outcomes relevant to this review:** **Improvement in vertigo** Vertigo was assessed by the participants themselves using a 5‐point system (0 to 4, with higher scores representing greater improvement: 0 = no improvement 1 = mild improvement, 2 = moderate improvement, 3 = excellent improvement and 4 = completely asymptomatic). This scoring system was dichotomised, so that scores of 0 to 2 represented 'little improvement' and scores of 3 or 4 represented 'marked improvement'. No validation of this scoring system was reported. **Change in vertigo** This outcome was not reported **Serious adverse events** Not fully reported. The authors do report on other adverse effects and state, "These side effects were not significant enough for the subjects to stop the study mid‐way", therefore we may presume that no serious adverse events were identified, but it is not clear whether these were systematically assessed and recorded. **Secondary outcomes relevant to this review:** **Disease‐specific health‐related quality of life** This outcome was not reported **Improvement in headache** Improvement in headache severity was assessed using the same scoring system as for vertigo (0 to 4, with higher scores representing greater improvement). It is not clear if this captures only the intensity of headache, or is a global score of severity (a separate scale was also reported for frequency of headache). **Improvement in other migrainous symptoms ** Not reported **Other adverse effects** Sleep disturbance (somnolence) and weight change (weight gain) were reported. The authors did not report on the other adverse effects we had prioritised for this review. **Other outcomes reported in the study:** Vertigo frequency Headache frequency	
Funding sources	The study was supported by funding from the Fluid Research Grants, Christian Medical College Research, based in Vellore	
Declarations of interest	The authors declare that they have no conflicts of interest	
Notes	**Research integrity checklist:** No retractions or expressions of concern The trial was retrospectively registered (CTRI/2012/07/002779) No concerns over baseline characteristics of participants, although limited data are reported Plausible loss to follow‐up was reported, considering the trial was of short duration No implausible results are reported Blocked randomisation was used, which accounts for similar numbers allocated to each group	
***Risk of bias***		
**Bias**	**Authors' judgement**	**Support for judgement**
Random sequence generation (selection bias)	Low risk	Quote: "Patients were subsequently block randomized (blocks of 4) into two treatment arms, A and B using computer‐generated random numbers." Comment: computer‐generated randomisation.
Allocation concealment (selection bias)	High risk	Quote: according to the trial registration, "Method of Concealment: An Open list of random numbers." Comment: it appears that an open list of random numbers was used, therefore allocation could not be concealed.
Blinding of participants and personnel (performance bias) All outcomes	High risk	Comment: no blinding is described, open‐label trial.
Blinding of outcome assessment (detection bias) All outcomes	High risk	Comment: no blinding is described, open‐label trial.
Incomplete outcome data (attrition bias) All outcomes	Low risk	Quote: "One patient from Arm A and three patients from Arm B were lost to follow‐up even after numerous attempts were made to contact them both via telephone as well as by post" Comment: few dropouts and insufficient to affect conclusions of the study, although noted that dropout was higher in the no treatment group.
Selective reporting (reporting bias)	Unclear risk	Comment: although a trial registration was identified, this was retrospectively registered after completion of the study. Therefore we cannot assess whether the pre‐specified outcomes were fully reported.
Other bias	High risk	Comment: the methods used to assess improvement do not really look at ‘improvement’ versus none; there is also a fairly arbitrary cut off and we cannot ascertain the number that did not improve at all. This is also an unvalidated rating scale.

**Table:** Yuan 2016

***Study characteristics***		
Methods	Parallel‐group, 2‐arm, open‐label RCT with 3 months total duration of treatment and follow‐up	
Participants	**Setting: **single‐centre study, conducted in China. Participants were recruited from August 2013 to May 2014 **Sample size:** **Number randomised:** 27 participants **Number completed:** 23 participants **Participant baseline characteristics** **Age: ** Only reported for the entire cohort: mean 45.33 years (SD 6.84); range 35 to 60 **Gender: ** Only reported for the entire cohort: 4 males and 23 females **Probable/definite vestibular migraine: ** Diagnostic criteria stated in the article indicate that only participants with definite VM were recruited **Attack frequency at baseline: ** Only reported for the entire cohort: mean 7.26 episodes (SD 2.62) over a 3‐month period **Duration of disease:** Not stated **Inclusion criteria:** People with definite vestibular migraine according to the IHS/Bárány Society criteria (see Appendix 1). Aged between 18 and 65 years. Vertigo attacks at least twice per month in the preceding 3 months, or the number of cumulative dizziness days exceed 4 days (in the same time period), and the participant themselves is seeking preventive treatment. **Exclusion criteria:** Other causes of vertigo, including Ménière's disease, benign paroxysmal positional vertigo and vestibular neuritis. Other acute or chronic diseases (e.g. cerebral infarction, cerebral haemorrhage, multiple sclerosis) that may have caused vestibular symptoms. People with hypertension who were receiving treatment with calcium channel blockers. **Diagnosis of vestibular migraine:** IHS/Bárány Society criteria (see Appendix 1)	
Interventions	**Intervention (n = 13 randomised, n = 12 completed):** oral flunarizine hydrochloride, 10 mg once daily, to be taken at night **Comparator (n = 14 randomised, n = 11 completed):** no treatment **Background interventions administered to all participants:** both groups received 12 mg betahistine 3 times per day for 48 hours during a symptomatic vertigo attack. All participants were advised to reduce stress, take aerobic exercise, have a regular sleep pattern, “control their emotions” and avoid foods that may induce attacks.	
Outcomes	**Primary outcomes relevant to this review:** **Improvement in vertigo** Assessed as the number of participants in whom the frequency of vertigo episodes improved by the end of treatment **Change in vertigo** Reported as the frequency of vertigo episodes over the 3‐month period of follow‐up **Serious adverse events** Some adverse events are reported; none of these are stated to be serious. We assume this means that no serious adverse events occurred, but cannot be certain of this. **Secondary outcomes relevant to this review:** **Disease‐specific health‐related quality of life** Not reported **Improvement in headache** Not reported **Improvement in other migrainous symptoms ** Not reported **Other adverse effects** Sleep disturbance and weight change were reported **Other outcomes reported in the study:** No additional outcomes are reported	
Funding sources	No funding is reported	
Declarations of interest	There is no declaration regarding the potential for competing interests	
Notes	**Research integrity checklist:** No retractions or expressions of concern No registered protocol was identified for this trial Baseline characteristics for the separate groups were not reported, therefore we are unable to determine whether there are extreme differences or similarities between the 2 groups Plausible loss to follow‐up was reported No implausible results are reported Slightly different numbers of participants were recruited to each group (13 versus 14)	
***Risk of bias***		
**Bias**	**Authors' judgement**	**Support for judgement**
Random sequence generation (selection bias)	Low risk	Quote (translation): "In EXCEL, the formula = ROUND (RAND () ×50+0,) was used to produce the rounded random number between 0 and 50, which was saved by a non‐receiving physician (data manager). After confirming the inclusion of the patient, the receiving physician contacted the data manager to obtain the random number of the patient and determine the treatment plan. They were divided into two groups according to odd and even numbers, and those with odd numbers were assigned to group A and those with even numbers to group B." Comment: computer‐generated randomisation sequence.
Allocation concealment (selection bias)	Unclear risk	Comment: insufficient information to assess whether allocation concealment was adequate.
Blinding of participants and personnel (performance bias) All outcomes	High risk	Comment: open‐label trial, no blinding was used.
Blinding of outcome assessment (detection bias) All outcomes	High risk	Comment: open‐label trial, no blinding was used.
Incomplete outcome data (attrition bias) All outcomes	High risk	Comment: dropout of 22% in control group compared to 8% in intervention group, sufficient to affect the results.
Selective reporting (reporting bias)	Unclear risk	Comment: no registered protocol was identified.
Other bias	Low risk	Comment: no other concerns were noted.

BP: blood pressure; DHI: Dizziness Handicap Inventory; IHS: International Headache Society; NSAID: non‐steroidal anti‐inflammatory drugs; RCT: randomised controlled trial; SD: standard deviation; VM: vestibular migraine

### Characteristics of excluded studies [ordered by study ID]

**Table:**

Study	Reason for exclusion
ACTRN12616000683437	This is not an RCT. It is trial registration for a non‐randomised study assessing the use of cervical and ocular vestibular evoked myogenic potential testing for diagnosis of vestibular migraine.
Byun 2021	This is a systematic review. The reference list has been checked to ensure that any relevant studies have been included.
ChiCTR1800014766	This is not an RCT. It appears to be a prospective cohort study to assess the clinical features of people with vestibular migraine.
CTRI/2019/09/021185	This is not an RCT. It is a non‐comparative study, where all participants will receive flunarizine.
Gode 2010	This is an RCT, but participants were randomised to receive either low‐dose (50 mg daily) or high‐dose (100 mg daily) topiramate. Therefore the comparator is not appropriate for this review.
Gordon 1993	This is a cross‐over RCT, but the population included had headache migraine, not vestibular migraine.
Liu 2017	This is a randomised trial, but no participants received placebo or no treatment. Allocation was to one of three interventions: venlafaxine, flunarizine or valproic acid.
NCT00732108	This is a trial registration for a planned RCT of people with migraine‐associated dizziness, comparing topiramate with placebo. The details from the registration site indicate that this study would have been eligible for inclusion in the review. However, the study was withdrawn prior to enrolment of any participants.
NCT03578354	This is a trial registration for a planned RCT. The details on the registry site indicate that this study would have been eligible for inclusion in the review. However, the study was withdrawn due to a lack of funding, prior to the enrolment of any participants.
NCT05472675	The comparator is not relevant. The intervention (local anaesthetic and botulinum toxin) will be compared to people receiving beta‐blockers.
PACTR201909600414183	This RCT compared two interventions (cinnarizine and topiramate), without the use of a placebo/no intervention arm.
Salviz 2015	This is an RCT but no group received either placebo or no intervention. The comparison is only between venlafaxine and propranolol.
Staab 2015	This RCT compares verapamil and sertraline; there is no group that received no intervention or a placebo.
Wang 2020	This is a systematic review. The reference list has been checked to ensure that any relevant studies have been included in this review.

IHS: International Headache Society; RCT: randomised controlled trial

### Characteristics of ongoing studies [ordered by study ID]

**Table:** NCT04417361

Study name	'Galcanezumab for vestibular migraine'
Methods	This is a parallel‐group, randomised, controlled study with 3 months duration of treatment and follow‐up
Participants	Estimated enrolment: 50 participants **Inclusion criteria:** Males and females aged 18 to 75 years Diagnosis of probable or definite vestibular migraine, according to the IHS and Bárány Society criteria Written, informed consent Baseline and study visit 2 VM‐PATHI score > 25 Baseline "definite dizzy days" > 4 from month 0 to month 1 Fluent English speaker 80% adherence or better to daily text messages during baseline phase Access to email and mobile phone **Exclusion criteria:** Pregnant, breastfeeding or unwilling to use birth control during study participation Presence of a condition that would compromise the safety of the patient or the quality of the data Allergy to, or prior treatment with, galcanezumab History of ear surgery, other than ventilation tubes Other vestibular diagnosis (excluding BPPV) Failure of treatment with > 4 prophylactic migraine medications History of serious medical or psychiatric disease
Interventions	**Intervention:** galcanezumab will be self‐administered using a preloaded syringe; 240 mg will be administered in month 1, followed by 120 mg per month during months 2 and 3 **Control:** placebo will be supplied in preloaded syringes and administered with the same regime as the active intervention arm
Outcomes	Change in VM‐PATHI (Vestibular Migraine‐Patient Assessment Tool and Handicap Inventory) between baseline (month 0) and after treatment (month 4) Change in the number of "definitive dizzy days", measured daily and reported as the change from baseline to month 4 Percentage of participants in each arm who experience a 100%, 75%, 50%, 25% or 0% reduction in "definitive dizzy days" to month 4 Change in Dizziness Handicap Inventory score from baseline to month 4 Change in Patient‐Reported Outcomes Measurement Information System Short Form (PROMIS SF) v1.2 ‐ Global Health Scores from baseline to month 4
Starting date	18 September 2020 Estimated study completion date 1 July 2023
Contact information	Jeffrey Sharon: jeffrey.sharon@ucsf.edu Adam Gardi: adam.gardi@ucsf.edu
Notes	This trial will be conducted in collaboration with Eli Lilly and Company

BPPV: benign paroxysmal positional vertigo; RCT: randomised controlled trial; SD: standard deviation; VM: vestibular migraine; VM‐PATHI: Vestibular Migraine Patient Assessment Tool and Handicap Inventory

## Differences between protocol and review

We planned to use the Trustworthiness Screening Tool from Cochrane Pregnancy and Childbirth to identify studies for inclusion in the main analysis (Webster 2022c). However, as described in Selection of studies, we did not exclude studies from the main analysis on the basis of concerns whilst using this tool. We considered that the overall certainty of the review findings (all very low‐ or low‐certainty) would not be impacted by this decision. 
